# The MPI Facial Expression Database — A Validated Database of Emotional and Conversational Facial Expressions

**DOI:** 10.1371/journal.pone.0032321

**Published:** 2012-03-15

**Authors:** Kathrin Kaulard, Douglas W. Cunningham, Heinrich H. Bülthoff, Christian Wallraven

**Affiliations:** 1 Department of Human Cognition, Perception and Action, Max-Planck Institute for Biological Cybernetics, Tübingen, Germany; 2 Brandenburgische Technische Universität Cottbus, Cottbus, Germany; 3 Department of Brain and Cognitive Engineering, Korea University, Seoul, South Korea; Bielefeld University, Germany

## Abstract

The ability to communicate is one of the core aspects of human life. For this, we use not only verbal but also nonverbal signals of remarkable complexity. Among the latter, facial expressions belong to the most important information channels. Despite the large variety of facial expressions we use in daily life, research on facial expressions has so far mostly focused on the emotional aspect. Consequently, most databases of facial expressions available to the research community also include only emotional expressions, neglecting the largely unexplored aspect of conversational expressions. To fill this gap, we present the MPI facial expression database, which contains a large variety of natural emotional and conversational expressions. The database contains 55 different facial expressions performed by 19 German participants. Expressions were elicited with the help of a method-acting protocol, which guarantees both well-defined and natural facial expressions. The method-acting protocol was based on every-day scenarios, which are used to define the necessary context information for each expression. All facial expressions are available in three repetitions, in two intensities, as well as from three different camera angles. A detailed frame annotation is provided, from which a dynamic and a static version of the database have been created. In addition to describing the database in detail, we also present the results of an experiment with two conditions that serve to validate the context scenarios as well as the naturalness and recognizability of the video sequences. Our results provide clear evidence that conversational expressions can be recognized surprisingly well from visual information alone. The MPI facial expression database will enable researchers from different research fields (including the perceptual and cognitive sciences, but also affective computing, as well as computer vision) to investigate the processing of a wider range of natural facial expressions.

## Introduction

Faces are one of the most ecologically important stimuli of visual perception. Over the last decades, perceptual and cognitive studies have repeatedly shown that humans are remarkably good at recognizing face information like gender, age, identity and facial expressions. Facial expressions are special inasmuch as they constitute the only information in the face that - besides mouth movements for visual speech - rapidly and constantly changes in a variety of complex ways. We are, however, easily able to tell different expressions apart within only a short glance. Moreover, in order to extract the correct meaning of the different types of facial expression, we do not necessarily need to know the person; that is, facial expression processing seems largely invariant to facial identity ([1], [2], but see also [3]–[5]). With applications not only in the perceptual and cognitive sciences, but also in affective computing and computer animations, it is not surprising that facial expression research has gained lot of attention over the last decades.

Compared to other species, humans have developed highly sophisticated communication systems for social interactions. In 1970, Bridwhistell demonstrated that during a typical communication, the verbal components convey one-third and the non-verbal components two-thirds of social meaning [6]. In addition to body gestures, facial expressions are one of the main information channels in non-verbal interpersonal communication [7]. Given their importance for non-verbal communication, facial expressions contain a dual aspect: they carry emotional meaning, and they also serve as a communicative information channel. This fact was already stated by Darwin in his seminal work on facial expressions ([8]/(1872) see also [9], [10]).

Interestingly, despite this dual aspect, expressions of emotion are by far the most well-studied component of facial expressions and thus represent the aspect that is best understood [11]–[13]. Previous research suggested that among emotional expressions, there exists a small number of “generic” or “universal” expressions. These “universal” expressions (usually defined as happiness, sadness, disgust, surprise, fear, and anger - but see [14]) are recognized well and interpreted similarly across many cultures ([15], [16], but see [17]–[19]). Although emotional expressions represent an individual's internal state, it is assumed that they also partly arise through interaction with others [20]. Hence, emotional expressions also have an important social function in interpersonal communication [21].

Despite the strong communicative aspect of facial expressions, however, there is a tendency to equate facial expressions solely with emotional expressions [22]. The following lists several more encompassing definitions of the different classes of facial expressions that help to stress their complex, dual nature: Fridlund, for example, differentiated facial expressions into three different classes: (1) purely reflexive facial postures and movements, (2) apparently emotional expressions, and (3) paralinguistic or communicative facial expressions (including, for example, a confirming head nodding, hence these expressions are related to speech) [9]. Facial expressions can also be discriminated by the actual mental state of the sender: there are facial expressions referring to a cognitive mental state (e.g. thinking, deciding or planing) and facial expressions referring to an emotional mental state [23]. Bavelas and colleagues distinguished between facial expressions of emotion and socially oriented facial displays [24]. Moreover, Fridlund shared the view that communicative facial expressions are the most often used expressions [25]. This is in line with Ekman since this study proposed that only a minority of facial movements do reflect emotional expressions ([12] see also [26], [27]).

Given the dual nature of facial expressions, few theoretical attempts have been made to disentangle the commonalties and differences of emotional and conversational facial signals. Ekman suggests that the commonalities of both expression classes lie in the fact that both need to occur in a conversation and hence both types of expressions require the presence of a second person [12]. Ekman also suggested the following differences between emotional and conversational signals: 1) coherence and stage of occurrence in life: emotional expressions develop earlier than purely conversational signals; in addition, emotional signals are more coherent than the majority of conversational signals, 2) speech influence: emotional expressions develop prior to speech, whereas conversational signals needs at least basic proficiency in intentional spoken language, 3) difficulty of performance: compared to emotional expressions, (at least some) conversational expressions are easier to perform, 4) complexity of the subject to be referred to: conversational signals that do not rely on language may refer to less complex subjects than emotional signals, 5) social inhibition: feeling unobserved elicits the occurrence of emotional expressions which does not hold for conversational signals, 6) affective state: in a conversation, emotional expressions rely more on the affective content and consider the feelings of the speaker and listener, 7) facial behavior differences: both expression categories might rely on the same visible muscle activation but differences might be obtained in the onset, duration and offset of this muscular change, and 8) universality: some emotional expressions are thought to be recognized across many cultures, whereas this might not hold for conversational signals. With respect to the speech-related differences between emotional and conversational expression, Bavelas and Chovil also stated that although emotional expressions are important for social interaction, it is assumed that they have stereotypic forms that are virtually independent of the linguistic content [22]. Moreover, they assumed that the meaning of further nonverbal but visible acts in a conversation depend rather on the linguistic content and that these acts are neither intrinsic nor isolated. Despite the long list of potential differences between emotional and conversational expressions that have been theoretically elaborated on, strong empirical evidence for these differences, however, is missing to date.

To facilitate the detailed investigation of the complex space of facial expressions, it is necessary to have access to a well-defined database that - in addition to the emotional expressions - also includes facial expressions with a communicative purpose. The current databases available to the community, however, primarily include a small number of emotional expressions (see Table S1). One noteworthy exception is a commercially available database which contains roughly 400 different expressions [28]. The expressions are grouped into 24 categories and are displayed by six different people of different age. The database was developed to teach people with autism spectrum disorders to recognize and understand emotions. To our knowledge this database has not been used for the study of human communicational abilities or perceptual processing of facial expressions.

Nevertheless, there are a few prior studies that also included some conversational expressions: For example, in studies on sign language, it was found that both syntax and lexical information are encoded by the face [26], making these so-called linguistic facial expressions critical for clear interpretation of what is being signed. These expressions differ from emotional expressions in their scope, timing, and in the facial muscles that are used. Furthermore, they have a clear on- and off- set and are highly coordinated with specific parts of the signed sentences [29]. McCullough and Emmorey investigated the categorical perception of two linguistic and two emotional facial expressions in normal and deaf participants [29]. Although the normal group was not experienced with linguistic facial expressions, a categorical perception was also found for those expressions, thus indicating similar processing mechanisms for both linguistic and emotional expressions. In perhaps the most detailed investigation of both emotional and conversational expressions in the context of perceptual research, Nusseck et al. investigated the influence of different face areas on recognition accuracy [30]. The emotional expressions tested were happy, disgust, sad, and surprised, and the conversational expressions were agreement, disagreement, thinking, confusion, and clueless. Overall, both expression categories could be reliably recognized. In addition, the authors found a complex pattern of face areas that were important for recognition, for example, the eyes were sufficient for the expressions thinking and clueless, whereas recognizing the expression confusion additionally needed mouth information.

Studies about facial expression are not only of interest in social and clinical psychology and psycholinguistics. Computers have now pervaded most aspects of our daily life, and one major aim in computer science is to realize and optimize this human-computer interaction. The design of human-computer interfaces has turned away form computer-centered to human-centered designs inasmuch as the latter takes important communicational aspects into account [27]. This idea goes back to Nickerson who first summarized typical characteristics of human-human interaction that might be considered in the interaction with computers [31]. At that time, the aim was to create analogues between a human nonverbal communicational signal and the respective computer signal. Nowadays, one important aim of human-computer interfaces is to create “embodied conversational agents”, that is, agents exhibiting the same communicational skills as humans in face-to-face conversations. These properties include for example the ability to recognize and respond to verbal and non-verbal input and output, as well as the ability to deal with conversational functions including recognizing and displaying conversational signals [32]. Therefore, Cassell suggests that the development of successful agents should be based on the study of human interaction [32]. As it is known from social psychology, facial expressions can be used to control the flow of conversations [7], [33]. Two fundamental characteristics in this context are turn-taking and back-channelling signals. According to Ynge, back-channelling signals convey an understanding of what has been said along with no interruption of the ongoing conversation [34]. In contrast, turn-taking refers to the change of speakers in a conversation following a specific signal. Since facial expressions have the highest impact on a conversation ([35] suggests, that facial expressions constitute up to 55% of a conversation) Pantic and Rothkrantz conclude that considering facial expressions in human-computer interfaces enables a stronger and hence more efficient interaction [33].

In order to design believable and effective communicative agents, the automatic *analysis* of facial expressions is crucial so that the agent might understand and react appropriately to the human. In the field of automatic expression classification, two approaches have been established [33]: 1) using visible facial muscle activation, or 2) using prototypical facial expressions. Similarly to the existing bias on emotional expressions in behavioral research, most existing systems are also used for classification of emotional expressions. As reviewed, for example, in Brave and Nass, the average accuracy of systems ranges from 90%–98%, indicating a high degree of success in recognizing emotional expressions [36]. These systems have only been tested, however, on prototypical rather than natural expressions [36], [37]. Although there have been attempts to build realistic embodied agents (see also [38]–[40]), state-of-the-art in human-computer interaction is the ability of computers to recognize *what* is being said, but not *how* things have been said [41]. For the latter, not only are accentuation and prosody important, but a deeper understanding of the non-verbal, visual communication signals becomes essential. Since computers do not have the ability to recognize for example pleasantness, annoyance, interest or boredom, human-computer interaction quickly becomes inefficient if not awkward [41]. Recent advances in computer vision have made some progress in terms of automatic recognition of dynamically presented emotional expressions (see [42]), however, the field is very much in its infancy as interpretation of conversational signals is concerned. Interestingly, despite the sentiment in this field that the design of successful conversational agents should be based on the study of human conversational behavior [43], relatively little is actually known about the perceptual and cognitive processing of conversational expressions. Finally, comparing existing systems is challenging given that there is no standardized database that includes natural emotional and conversational expressions displayed statically or dynamically and at different views ([33], for a review on databases used for computational research on emotions see [44]) as standardized databases in this field are normally used for testing algorithms allowing automatic classification.

As suggested by Ekman, in order to understand human communication, one must both understand the conversational expressions as well as the emotional expressions [12]. The vast majority of studies investigating emotion recognition of facial expressions uses stimuli that have before been evaluated according to their physical properties. The standard for this is based on muscular activity leading to face distortions. This idea goes back to the work by the French physician Guillaume-Benjamin Duchenne, who pioneered the science of muscular electrophysiology. Duchenne elicited facial expressions through electrical stimulation of particular face muscles (see the well-known “Duchenne smile”, [45]). In these studies, the ground-truth information, that is the source causing the expression, is given by the physical deformation of the face muscles. However, the muscle deformation does not give the *cause* of the facial expression. With respect to social interaction and in particular to communication through facial expressions, the cause for the expression is relevant since, for example, it could be a turn-taking signal in communication. How can one define a facial expression by the cause leading to that particular face deformation? One possibility is the so-called “method-acting protocol” commonly used in actor training. Here, actors are given particular background scenarios that are thought to elicit the respective facial expression. Thus, the background scenario in these cases represents the ground-truth information of the respective facial expression. To our knowledge, there are only few databases available in which a “method-acting” protocol was used in order to elicit the desired emotional facial expression ([46]–[52]).

Furthermore, most studies use *photographs* of emotional facial expressions as stimuli, thereby restricting the potential studies to static expressions only (only approximately one third of the reviewed databases in Table S1 contain dynamic stimuli). However, the world around us is highly dynamic and there is evidence pointing towards an advantage of facial motion in recognition of emotional expressions. In the pioneering work of Bassili, both point-like videos of expressions and the corresponding static image at the apex of the expression were used [53]. Moreover, static, normal stimuli were also included. Interestingly, the study was able to demonstrate a clear recognition advantage for point-like videos over static stimuli. This advantage was even enhanced when using normally illuminated videos of those expressions. Wehrle and colleagues showed that emotions were less often confused when dynamic information was available [54]. Ambadar et al. investigated the effect of motion on subtle facial expressions using different scrambling procedures, finding a clear dynamic advantage for emotional expressions that is due to the role in perception of changes [55]. Using morphing sequences of emotional facial expressions, Kamachi et al. found that it is rather the speed and not the duration that influences the perception of expressions [56]. Similarly, it seems that the visual system is especially sensitive to the dynamics in the early stages of an expression [57]. Whether a movement is sufficient or necessary for recognizing particular expressions was examined in Nusseck et al. [30]. They demonstrated that movements of different face parts contribute differently to expression recognizability. Thus, although most often static images of emotional facial expressions have been used, there is behavioral evidence pointing towards a dynamic advantage in facilitating recognition accuracy of emotional facial expressions. However, whether or not this includes all kinds of expressions similarly, remains unclear: Harwood and colleagues, for example, found an advantage of dynamic information only for the emotional expressions sad and angry [58]. Similarly, Fujimura and Suzuki showed that the beneficial effect of dynamic information depends on the emotional properties of facial expressions [59]. Detailed experiments in Cunningham and Wallraven showed a dynamic advantage for recognizing sad and surprise, but not for happy for which the static presentation was better [60].

The difference in processing of facial expressions displayed either statically or dynamically is also found in neuroimaging data with different neural activity patterns for static and dynamic facial expressions: Kilts et al. found dissociable neural pathways in the recognition of emotion in static and dynamic facial expressions [61]. Also, several brain imaging studies show an enhanced neural activity when using dynamic facial expressions (e.g. [62], [63]). Fox et al. were able to demonstrate that compared to static images of faces, videos of moving faces more strongly activate all face selective regions in the human brain using functional MRI ([64], see also [65]). Thus, in addition to behavioral studies, neuroimaging studies also suggest an advantage of dynamic information in facial expression processing. The importance of dynamic information in face processing has also been considered in the face processing model by O'Toole and colleagues [66]. Hence, although the vast majority of studies has used static images of emotional facial expressions, there is both behavioral and neurophysiological evidence for differences in processing of dynamic (emotional) facial expressions. Since dynamics form such an important component of facial expressions, we need to extend existing studies to a more comprehensive and representative sampling of the spatio-temporal information in faces. This requires new databases containing not only static but also dynamic examples of a broad variety of facial expressions, such as the one presented here.

To summarize, although theoretical attempts have been made to differentiate between the two expression categories of emotional and conversational expressions, detailed experimental studies on their commonalities and differences are missing so far. Here, we present the MPI facial expression database as a new resource to the community that contains both emotional and conversational expressions. Moreover, since emotional expressions are already recognizable in a static image, the database is available in two versions: a static and a dynamic version. In order to achieve a compromise between control and naturalness, the expressions have been recorded based on the method acting protocol. This means that the expressions contained in the database are defined by the method-acting scenarios, and hence by the *context* rather than by the physical face deformation. In addition to describing the recording protocol and the database in detail, we also present results from a validation experiment of the database.

## Methods

### Ethics Statement

The expression database and the validation experiment described later in this manuscript use human volunteers. Informed written consent was obtained prior to any experiment or recording from all participants. Participants and data from participants were treated according to the Declaration of Helsinki. The recording methods of the database and the subsequent validation experiment were approved by the local ethics committee of the University of Tübingen (Project number: 89/2009BO2).

### Development of the facial expression database

In the following, we will describe the MPI facial expression database in more detail: including the choice of expressions that were included, the recording protocol and models, the post-processing, as well as additional features included with the database (audio recordings and 3D scans).

#### Determination of facial expressions to be recorded

As stated above, one of the major goals of the database is to capture both emotional and conversational expressions. In order to provide a more fine-grained resolution, the database was designed with three levels of hierarchy: (1) basic-level emotional expressions, (2) basic-level conversational expressions (please note that “basic-level” in this content encompasses expressions as for example “agreement” or “sadness”, whereas “reluctant agreement” for example would correspond to a subordinate expression. We do not claim that the basic-level expressions share all aspects of the original definition presented by Rosch and colleagues [67].), and (3) subordinate expressions. In addition, for each expression we determined a corresponding context scenario that could be used to elicit the expressions.

Basic-level emotional expressions convey information about the emotional state of the sender and are thought to be the origin of all other emotional expressions (e.g [68], [69]). Hence, these expressions represent categories of expressions with each category being based on similar emotional states [70]. Similarly, conversational facial expressions are defined as expressions that primarily supply the sender with communicative information - again, basic-level conversational expressions represent broad categories of similar communicative signals (such as thinking, agreeing, etc.). Through modification, humans are able to form more complex, qualified, or even mixed emotional and conversational expressions (such as a sad smile or a considered agreement, for example) - these expressions then belong to the subordinate-level facial expressions.

For the range of facial expressions to be recorded in the database, we considered the well-known basic emotional expressions happiness, sadness, anger, fear, and disgust. In order to cover a large range of subordinate emotional expressions, our selection of those expressions was based on the study by Shaver et al. in which subordinate categories of basic emotions were identified [71]. These include, for example, impressed, contempt, as well as pride - the corresponding context scenarios that were selected for these expressions were “You observe someone dancing and think: Wow, that's really good!”, “You think of someone you despise.”, and “You have reached a goal and you are happy to have accomplished it.”. Note, that the connection between the labels and the context scenarios of course still needs to be validated.

With respect to conversational expressions, we included expressions that span a large range of different expression categories motivated by the research of Pelachaud and Poggi [72]. Here, communicative functions of facial expressions are clustered into five groups providing information about 1) the location and properties of objects or events, 2) the degree of certainty, 3) the intentions, 4) the affective states, and 5) metacognitive information on the mental action. Particular, the third class - the intentions - allow a distinction of facial expressions that are intended to express the goal of the sender. These general goals can be broadly categorized into a request, an information, or a question. Furthermore, within each category, specific performatives are to be distinguished: a request might be given in form of a proposal or an order; an information might be an announcement or an assertion; questions might be given in form of a leading question or informative question. With the study by Pelauchaud and Poggi in mind, we considered everyday situations that, on the one hand, differ with respect to their overall goal. As an example, the scenario “What did you just say?” is based on a question, whereas “I'm impressed by the way how you dance.” communicates an information. In contrast, there are also everyday situations that differ in the addressee, for example, “I feel sorry for you” versus “I'm annoyed”. Moreover, there are everyday situations that can be used to distinguish the degree of certainty of the sender: thinking about what you had for breakfast yesterday is easier - and therefore more certain - to answer than if you are asked to name the president of a far-away country. Finally, we considered everyday situations in which the power relation of the sender differs: For example, in an arrogant facial expression (such as might be elicited in the situation “Only I am the best!”), the sender acts more dominantly, than when communicating “I can follow what you are saying, please continue.”. At this point, it should be noted that so far empirical evidence for their relevance exists only for a few of these conversational expressions. While it seems easy to list many expressions, it will be necessary to investigate the validity of the conversational expressions - a part which will be covered later in the validation study.

Taken together, the database is based on a large range of different everyday situations that are designed to elicit facial expressions. In our case, the outcome - the facial expression itself - is defined by the everyday situation, that is, its communicative or emotional context; hence, the expressions included in the database contain ground-truth information regarding their occurrence in everyday-life (see also [73]). This is the first time that such ground-truth information is available.

An overview of the 55 different everyday situations can be found in Table S2. In this table, we also summarized each everyday situation by roughly naming the associated facial expression together with a broader classification of the expressions' type. However, it should be noted that at this stage, the validity of these labels and the classification of the expression type is not yet tested - validation studies on both the labels and the resulting expressions are reported later.

#### Expression models

Twenty native German (ten female) participants all of whom had no professional acting experience took part in the recordings. In the following, we refer to these participants using the term *model* to clarify that they are not professional actors. Due to technical problems the recordings of one male model had to be excluded. Participants were compensated at standard rates of 8 Euros per hour for their time.

Only native German participants took part in order to exclude possible cultural influences in producing facial expressions. Elfenbein et al. showed that cultural dialects exist in the production of posed facial expressions [74]. Moreover, cultural influences can be found at all levels of processing guiding social interaction [75].

In addition, we restricted the age-range of participants to lie between 20 and 30 years since in most studies participants are usually around 20. In face recognition research, it has been shown that participants identify a target face more accurately when the target is of the participant's age [76], [77]. In addition, there is growing evidence pointing towards an own-age bias also in facial expression recognition ([78], [79] - see also the FACES database, which includes emotional facial expressions covering different age ranges [80]).

In recording any expression database, one of the most fundamental decisions to be made is how the expressions should be elicited. Broadly speaking, there are two types of expressions: spontaneous and posed expressions [81]. Spontaneous expressions are defined as those that occur in real life, that is, spontaneous expressions are fully natural. In contrast, posed facial expressions - such as, for example, produced by professional stage actors - are assumed to be artificial [10] as they are often more proto-typed. Moreover, studies have shown that there are physical differences between the two expression classes: spontaneous smiles usually show smaller amplitudes with a more consistent relation between amplitude and duration than posed smiles do ([82], [83], see also [84], [85]). Interestingly, posed facial expressions are thought to be identified more easily compared to spontaneous facial expressions (e.g. [85]–[88]) - this might in part be due to an exaggerated intensity of posed expressions [89], similar to the caricature effect in face recognition [90]. Whereas it would be ideal to have fully controlled, spontaneous expressions, this goal cannot be achieved in reality due to lack of control over real-life situations. Many databases hence resort to professional actors - while this will produce very recognizable expressions, as stated above, actors sometimes tend to produce stereo-typed facial expressions. We therefore chose to record only participants without prior acting experience to try to capture more life-like, less prototypical expressions.

#### Material

**Figure 1:**
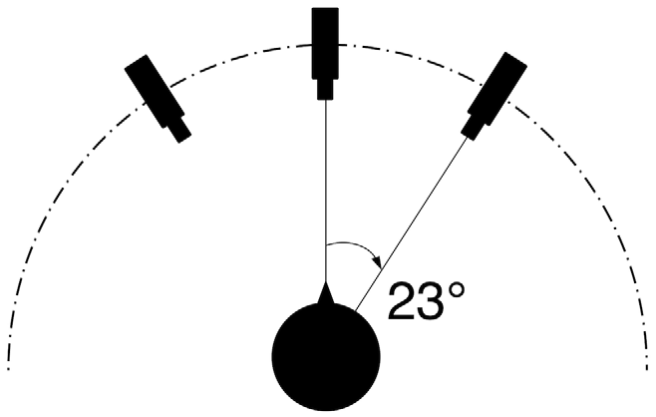
Set-up of the video lab. Figure shows the used set-up for expression recording. The expressions were recorded by three fully synchronized cameras. The models were sitting in front of the frontal camera and acted as if the central camera is a person to address. To facilitate this “face to face” scenario, the experimenter was standing behind the frontal camera.

The recordings were done with the Max Planck Institute for Biological Cybernetics's VideoLab (for more details see [91]), which is a custom-designed setup with six digital cameras arranged in a semicircle around the person to be recorded. The cameras are fully synchronized and have a PAL video resolution of 768×576 pixels. The expressions were recorded by three out of the six fully synchronized cameras: one frontal and two lateral views (on the right and left side, respectively) offset at an angle of 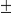
23 (see Figure 1). The cameras recorded at 50 frames/s and the exposure duration was set to 15 ms per frame. Lighting was provided by five high-frequency studio-lights carefully arranged to produce a flat lighting environment with as few cast shadows as possible. Because of the high driving frequency, the lights did not produce any noticeable flickering artifacts during the recording. Sound was not recorded.

**Figure 2:**
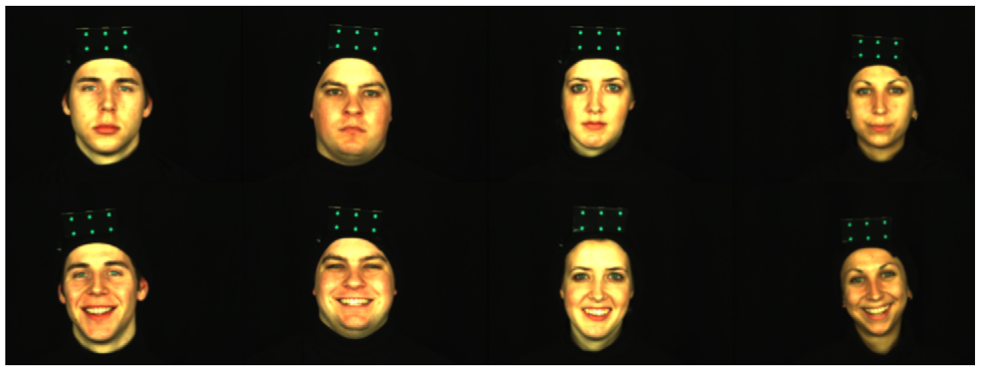
Example of models. Figure shows examples of four models out of the MPI facial expression database. Models were sitting in front of a black background wearing a black cloak. Moreover, they were wearing a black hat with six green markers on that worked as head tracking. The upper row shows the four models in a neutral position, whereas in the lower row models show a smile expression. Note that the expressions are available in a static and a dynamic version.

Participants were sitting in front of a black background wearing a black cloak. To aid the post-processing of the video sequences, the models wore a black hat with six green markers on that worked as head tracking markers (see Figure 2). Moreover, we controlled for all relevant camera parameters (focus, exposure, etc.), illumination settings, as well as the relative positions of the cameras.

#### Method

Each participant performed 56 different expressions acting as if the central camera was a person to address.

Following an approach commonly used in the field of acting, we based the recordings on a method acting protocol that has previously already been used in developing facial expression databases [46]–[52]. For our recordings, the participants were specifically told the everyday situations described in Table S2. They were then asked to remember a similar situation in their life, to imagine that they were in that situation again, and to act accordingly (with the exception that they were asked not to use their hands and not to speak). Participants were allowed to repeat the expression before the recording until they felt comfortable. With this method, we tried to elicit natural, yet controlled expressions. Furthermore, we took care not to provide an explicit facial expression label, but had the descriptions “speak for themselves”. This protocol tries to make the resulting expressions as natural as possible while still maintaing control over the expression context.

Each expression was recorded three times in a row with a little pause between repetitions in which the participant was asked to return to a neutral expression. In addition, we recorded each expression at two intensities: a lower intensity, which should correspond to the amount of emphasis in a standard conversation, and a higher intensity which should more strongly emphasize the content of the expression.

The recordings for each participant took place on two different sessions with approximately 4 hours used for recording the full set of facial expressions. The order in which the expressions were recorded was according to categories (negative and positive expressions, thinking expressions, etc.); however, because some expressions were easier to record than others, this order was modified slightly in some cases. In spite of that, in all cases participants were able to produce all expressions. The participants first produced the facial expression at high intensity followed by low intensity. Taken together, the new database contains approximately 20.000 video recordings (19 participants * 56 expressions * 2 intensities * 3 repetitions * 3 camera perspectives).

#### Post-processing of the video recordings

To enable in-depth investigations of the temporal context of facial expression processing, it is essential to create both a static and a dynamic set of expression stimuli. This requires to determine the peak-frame, or apex of the expression. Moreover, since the three repetitions of each facial expression were not separated during the recording, the begin- and end-frames of each expression recording needed to be determined. Note that the three cameras were time synchronized, such that a given frame number determines the same time point in each camera perspective obviating the need for manual synchronization.

The start, peak, and end frames of the recordings were defined as follows: The **begin frame** is the last frame of the dynamic facial expression at a neutral position of the face - just before the face starts to move. The **peak frame** is the frame of the dynamic facial expression in which the interpretation signal for the whole facial expression is presented as well as possible. The peak frame is therefore not defined as a feature position of face areas at a particular time. The **end frame** is the first frame of a video sequence of dynamic facial expressions after which the face returns to a neutral position.

With these definitions, each expression video contains a single facial expression that starts at a neutral position continues to a peak and returns back to a neutral position. The static stimulus only contains the peak of the expression.

For determining the begin-, peak-, and end-frames, we developed a user interface which allows to efficiently search and shuttle through the large number of recordings. The frames were determined for each camera perspective separately as it might be that a facial feature starts to move when not visible in the frontal camera perspective - as examples, lateral eye- or cheek-movements can sometimes be earlier detected at side-view camera perspectives.

#### Face Scan

The database also contains three dimensional face scans of most participants. These scans allow video manipulation techniques such as used in [30], [47], [91] as the rigid motion of the head can be reliably extracted from the markers participants wear.

Out of the 19 models who participated in the recordings of facial expressions, we asked 14 participants (nine female) to participate in the face scan. All participants gave written consent. For the 3D scan, models were asked not to put on makeup and not to wear clothing covering the neck. Moreover, male models were asked to be shaved on the day of the recording. All models were paid 6 Euros for the face scan.

The three dimensional face image of each model was captured using a Cyberware 3D Face scanner. Here, participants are seated on a chair in an upright position and asked to hold a neutral expression for 20 seconds. While scanning, they are asked not to move their eyes. To capture the image, a laser profile records the face while moving for one full rotation around the participant's head. The scanner records shape and texture of a face simultaneously and at the same resolution. Thus, each surface coordinate is registered with exactly one texture pixel (for detailed information see [92]).

#### Audio Recordings

The goal of the video recordings was to focus on non-verbal, purely visual facial expressions only. Thus, our models were asked not to speak while recording the expressions as mouth movements contain both expression and speech-related, visual information. Given that the expressions were chosen according to typical everyday conversations, it might be that the recognition of some expressions needs additional context information. In order to enable the investigation of multimodal processing (more specifically, the influence of language) on the recognizability of facial expressions, the database also includes audio footage that was recorded separately from the video footage.

For this purpose, typical German sentences for each everyday scenario used for recording the facial expressions were created. Those sentences were then spoken by 10 participants (5 female) out of the 19 participants who participated in the expression recordings - the participants were randomly selected. For the audio recordings, participants were seated in front of a condenser-microphone placed in a sound-proofed audio environment. The same method acting protocol as for the video recordings was used. Participants were asked to repeat the sentence three times in a row. The sentences were spoken at both high and low intensity corresponding to the intensities of the video recordings of the facial expressions.

### Validation of the new facial expression database

The concept of the MPI facial expression database is that it is based on everyday scenarios that are supposed to elicit facial expressions of different types. We still need to validate, however, that these everyday scenarios - the ground truth information for each expression - are, indeed, able to elicit clear and interpretable expressions. Hence, the first aim of our validation experiment is to validate the descriptions of the scenarios that form the basis of the method-acting protocol. The second aim of our validation experiment is to qualify both the visual recognition and the perceived naturalness of the video recordings themselves.

Taken together, our validation experiment was designed to validate both the input and the output of the expression database using two conditions. This experiment can therefore serve as a baseline for future experiments on processing of emotional and conversational facial expressions.

In the following we will describe the recruitment of the participants, the method, material and procedure for both conditions together, the analysis and results will be presented separately for each condition.

#### Population

The validation study was approved by the local ethics committee (this ethics proposal was certified together with the proposal for recording the database). In total, 20 native German participants (10 female) took part who were compensated at the standard rate of 8 Euros an hour for their participation. The participants were between 19 and 33 years old and had normal to corrected-to-normal vision. Participants suffering from deficits in interpreting facial expressions were excluded.

#### Method

A between-participants design was used to validate the new database. The experiment contained two conditions and participants were randomly assigned to either condition.

The first condition - the context-condition - aimed to validate the ground-truth information of the database. Ten participants (5 female) were asked to freely name the facial expressions that would be elicited given the *written* everyday situations that were used for the recordings. The answer was therefore solely based on the context information without any visual input.

The goal of the second condition - the visual condition - was first to quantify the visual perception of videos of facial expressions. Ten participants (5 female) who did not take part in the first condition and who did not know the models were asked to freely name the expression based on the video recordings of the database. In addition, participants were asked to rate the naturalness of each presented facial expression using a 5-point Likert scale. The second goal of this condition was therefore to directly assess the perceived naturalness of each expression.

In both conditions, participants' confidence in their naming answers was assessed using a 5-point Likert-type scale.

#### Material

The stimulus-set for the context condition consisted of 55 text descriptions of the everyday situations (ground-truth information) that were used for eliciting the facial expressions while recording the database. The expression “doe eyed” had to be excluded from this validation experiment since no scenario was available that did not also include the label of the expression.

For the visual condition, our aim was to validate the new database in a manner that would also serve as a starting-point for investigating conversational expressions in more detail. As the database contains a very large number of stimuli taking into account all expressions, models, views, and intensity levels, we decided to validate the database with a sub-set of all videos: more specifically, we used expressions of high intensity viewed from the frontal camera perspective from ten randomly chosen models (5 female). As the database contains three repetitions of each expression, we selected one repetition for each model during a pre-screening. Two of the authors and a further person selected the best repetition based on (subjective) performance quality and artefact-free recordings by majority voting. The final stimulus set for the visual condition included 51 out of the 55 expressions as some expressions were not well recorded for all of the ten models (these expressions include “anger”, “evasive”, “thinking remember neutral”, and “reluctant smile” - note, that this decision was only made to ensure a fully balanced design.)

#### Procedure

During the experiment, participants were seated in front of a standard 21-inch CRT monitor, on which the stimuli were presented in random order. For the context condition, the background color of the monitor was set to gray and text descriptions were shown in black to ensure comfortable reading. In the visual condition, the background color of the monitor was set to black as this blended well with the background color of the recordings. The display resolution of the monitor for both conditions was set to 1024×768 pixels.

In the context condition, participants first saw the text of the context scenario on the screen. The text was shown for the whole duration of the trial and only disappeared after participants entered their response. Similarly to the models during the recording of the database, participants were instructed to imagine a similar experience in their life and then to name the facial expression that would be elicited. They then had to describe the facial expression with a maximum of three words typed into a text field. No further restrictions were given as to the type of words (nouns, adjectives, etc.). After naming, we asked participants to indicate their confidence about the naming decision on a scale of one to five (1 = not confident at all, 5 = very confident) by typing the corresponding number into a text field. By pressing the button “save” all input fields as well as the scenario text disappeared and the next trial started. The experiment was split into two sessions, which took place on two consecutive days. Each session lasted about 1 1/2 hours. Participants were explicitly instructed to use the same strategies in both sessions.

As precise timing during playback of the video recordings was crucial, we used the Psychophysics Toolbox version 3 [93] in the visual condition. After loading the video sequence, the participant pressed a button for playback of the current trial. After the button press, a fixation cross was shown for 2 seconds at the place where the facial expression would be presented. This was always in the middle of the upper 2/3 of the screen. The facial expression was shown and disappeared immediately after playback. The lower 1/3 of the monitor was then used for participants' input. Participants had to name the facial expression in the same way as participants in the context condition. After naming, we asked the participants to indicate their confidence in each of their naming answers on a 5-point scale by typing the corresponding number into a text field. Furthermore, participants were asked to rate the naturalness of each observed facial expression by using a 5-point scale, with “1” indicating an extremely posed facial expression and “5” indicating a natural expression (as it would occur during a natural conversation). The visual condition also included the possibility to repeat the presentation of the facial expression by pressing a “repeat” button. After pressing the button, the input fields disappeared from the lower display area, the fixation cross was shown again for 2 seconds, and the current sequence was repeated, after which the input fields reappeared. The current trial was finished by pressing the button “Next expression”. After 30 trials, participants had the possibility to take a break. Due to the large number of stimuli (in total, 510 video stimuli per participant), the visual condition was split into several sessions, which took place on consecutive days. Each session lasted around 2 1/2 hours and the number of sessions was adjusted until all 510 stimuli were seen - for 9 participants this resulted in four sessions, one participant required five sessions. Participants were explicitly instructed to use the same strategies for naming and rating the facial expressions across all sessions.

#### Analysis and results for the context condition

*Free naming analysis:* One of the most relevant performance measures is whether the scenario descriptions (ground-truth information) for recording the new database elicit clear facial expressions. In order to do so, it is necessary to cross-validate the free-naming answers for each text description across participants. This task was done by three raters (one of the raters is an author of this paper), who classified the naming answers of participants into “valid” or “invalid”. “Valid” in this context was defined as an appropriate answer for the scenario description, which was provided to the raters. No further instructions on how to do this assignment were given.

For the analysis, we counted the number of valid and invalid answers. For calculating the latter, an answer was considered as being invalid as soon as it was declared as such by *one rater*, which represents a rather conservative measure of validity. Given this separation into valid/invalid, we assume that a given everyday situation with a high number of valid answers, indeed, is able to elicit a clear and interpretable facial expression. Conversely, we assume that everyday situations with a high number of invalid answers indicate that the scenario might generate ambiguous expressions. In addition, we report the usual inter-rater consistency as Fleiss Kappa.

The inter-rater consistency for our three raters was 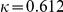, which represents “substantial” agreement according to the standard rating scale recommendations [94]. Given our conservative criterion for validity, overall, 81.09% of the 550 answers were rated as valid. When we relax this criterion to only two raters agreeing on the judgment, we obtain 88.18% valid answers - in the following, however, we will adopt the more stringent criterion. From these numbers alone and taken the complexity of the descriptions in the database into account, this is a very encouraging result as the vast majority of everyday descriptions indeed seem to elicit clear facial expressions.

**Figure 3:**
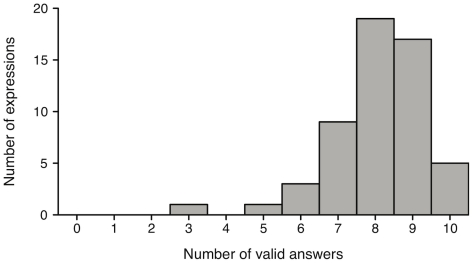
Context condition: Frequency of number of valid answers. Frequency distribution of the number of expressions with a given number of valid answers for the context condition. Maximum number of valid answers is 10 as there were 10 participants.

Figure 3 illustrates how many text descriptions were obtained for each number of valid answers. The clearly left-skewed distribution indicates that most text descriptions resulted in large numbers of valid answers. In contrast, only very few descriptions yield a low number of valid answers: there is only one scenario which has 3 out of 10 valid answers, another one has half of the answers being declared as valid, and 3 scenarios scored 6 out of 10 valid answers. In the following, we will discuss selected cases to give a more detailed interpretation of these results.

The scenario description “You observe someone dancing and think: Wow, that's really good.” was one of the text descriptions for which all answers were deemed valid. Overall, participants gave similar answers including for example “appreciating” or “admiring”. We can therefore confirm that this description does, indeed, elicit a clearly interpretable facial expression which might be labeled as “impressed”. The description “Someone is explaining something to you, but you don't understand.” belongs to the scenarios rated as highly valid with only two invalid answers: two raters rated “being at a loss” as an invalid answer and “not comprehending” was rated as inappropriate by one rater. All three raters agreed that answers like “asking” or “incomprehensible” were valid. Overall, we can therefore conclude that the corresponding scenario contains elements of “not understanding”.

One of the scenarios, for which we obtained more invalid answers is “Someone suggests something. You are really not sure about it, but in the end you disagree.”. More specifically, four answers were rated as invalid in this case. The majority of valid answers suggests “reluctant disagreement” to be the underlying facial expression. Two raters declared the answers “doubting” or “thoughtful” as invalid. These answers, however, still partly imply the suggested label of the expression with only the attribute “disagreement” missing. This example shows that the three raters were rather conservative in their criterion.

The most critical scenario is “After leaving your flat you realize you forgot to switch off the cooker.”. Here, 7 out of 10 answers were rated as invalid. A detailed analysis of this particular case shows that a large part of the high number of invalid answers is due to one of the three raters who declared four answers as being invalid. Expression names rated as being invalid were for example “upset” or “surprised”. The majority of answers still suggests “frightened” or “worried” to be the underlying facial expression. Because of the larger number of invalid answers, this scenario might cause problems, as the associated facial expression might not be clearly defined. It remains to be seen, how well the results of this scenario - that is the expression videos themselves - will be rated in the visual condition.

**Figure 4:**
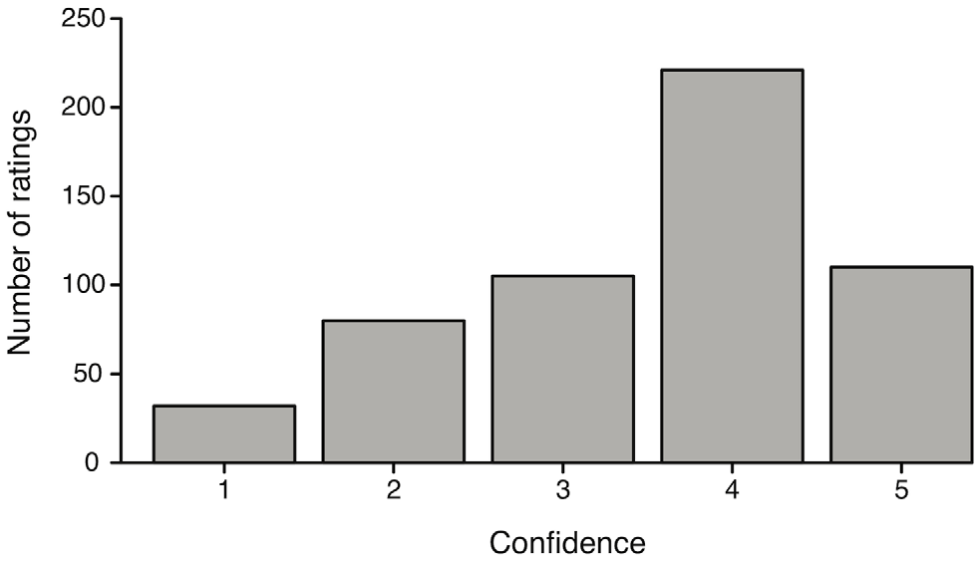
Context condition: Frequency of participants' confidence. Frequency distribution of participants' confidence ratings pooled over participants and expressions for the context condition. Confidence score 1 means “not confident at all” whereas 5 means “very confident”.

*Confidence ratings:* The individual confidence ratings were first analyzed by calculating the frequency for each of the five rating scores across all participants and text descriptions. The left-skewed distribution in Figure 4 shows that overall participants were confident about their naming answer (median = 4 with an interquartile range of 1). As usually found in rating experiments, participants less often used the more extreme values, however, 5 (indicating that participants felt most confident) was more frequently used than 1 (not at all confident). This confirms that participants felt rather confident in their free-naming answers. Out of all participants, only two showed generally lower confidence scores. For those participants, however, we did not obtain a higher number of invalid answers compared to the other participants, indicating a potential scaling or anchoring effect for their ratings.

Investigating the overall confidence ratings across participants for the text descriptions, we found that participants felt quite confident when descriptions were given that might elicit expressions like “remembering”, “not convinced”, “imaging”, “impressed”, “insecure”, or “don't hear”. In contrast, participants showed lower confidence ratings for expressions such as “bothering”.

Given that participants thought their answer to be perfectly valid for a particular text description, it might be especially useful to take their confidence ratings into account when analyzing the validity of naming answers. More specifically, one might assume that if all raters agree on the answer being inappropriate then the confidence rating for this particular answer would be low and vice versa. In order to measure whether there is a statistical difference of confidence ratings with regard to validity type, we first performed a Welch two-sample t-test. There was a significant effect for confidence ratings with valid answers receiving higher confidence scores than invalid answers (*t*(145.78) = 2.39, *p* 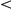.001). A post hoc power analysis, however, revealed that there was only a slight effect of confidence ratings on validity type (*d* = .28). This can also been seen in the following example: “You find moldy food in your fridge after you come home from a journey.”. Here, one participant was answering “I don't care” with a confidence rating of 5. This answer was declared as invalid by all three raters. After the experiment the participant told us that he is living in a shared flat and thus used to see moldy leftovers. Hence, whereas there is a slight correlation between the confidence and the validity judgments of the raters, the variability of the answers necessitates a closer look at individual outliers in order for the confidence answers to become useful.

Taken together, our first validation condition reveals that out of the 55 everyday descriptions, for 50 descriptions, 7 or more answers were declared as valid. Descriptions inducing expressions like “agree”, “disagree”, “lightbulb moment”, “bored”, “happiness due to achievement”, “impressed”, “remember”, “sad” or “tired” were examples of the most effective scenarios. We found only a few scenario descriptions that seemed to be interpreted ambiguously. Again, it remains to be seen whether the video recordings of facial expressions of those descriptions might also cause such an ambiguity. In addition, the confidence ratings confirmed that participants did have high confidence in their answers, with lower confidence ratings given in cases when the answer also had a higher probability of being rated as invalid. Whereas the confidence ratings differed between scenario descriptions, we found no description with extremely low confidence ratings.

Given the overall results of our free-naming analysis, we can confirm that our input - the scenario descriptions - are valid. They do elicit a variety of different types of facial expressions. Finally, having access to the naming answers of facial expressions we are able to label the ground-truth information according to the facial expressions they elicit in general. These labels were chosen such that they best summarize the given free-naming answers. We, however, do not claim that these are the most accurate labels, but they provide a good impression of both the names and meaning of the recorded facial expressions - especially for the previously less investigated conversational expressions.

#### Analysis and results for the visual condition

*Free naming analysis:* From ten participants we received a total of 5100 naming answers which were used for analyzing the naming performance in the visual condition. The analysis procedure here was the same as in the context condition. Moreover, the same three raters were asked for validating participants' free-naming answers.

Overall, approximately 60% of all answers were rated as valid. With again a substantial reliability of agreements between the raters when classifying the answers (Fleiss kappa 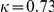), only 40% of the answers were declared as invalid.

**Figure 5:**
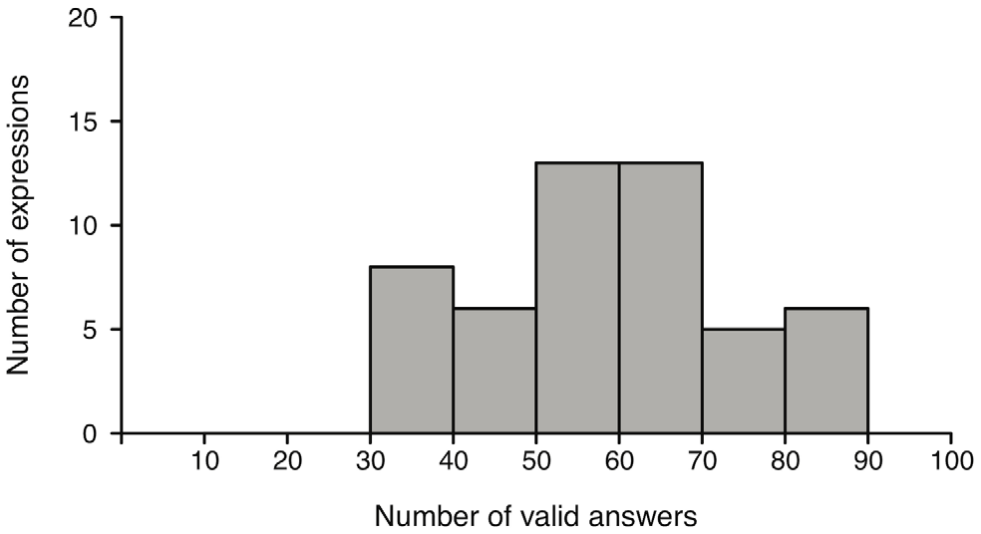
Visual condition: Frequency of valid answers. Frequency distribution of the number of expressions with a given number of valid answers for the visual condition. Since the maximum number of valid answers for each expression is 100 (10 models * 10 participants), expressions were grouped together resulting in group increments of 10.

In order to investigate the overall expression recognizability, we first summarized the number of valid and invalid answers over all ten models and ten participants for each expression. Note that each expression was shown by each of the ten models. Thus, if an expression of one model would not be recognized by all participants the number of invalid answers would be 10. If the expression shown by all ten models would not be recognized by all participants, the maximal number of invalid answers for this expression would be 100. Expressions were grouped together resulting in groups with increments of 10, and the total number of valid answers was calculated (see Figure 5). The total sum of number of expressions displayed in the figure is 51. Therefore, there is no expression performed by all ten models which receives only invalid answers. Overall, we obtain 6 groups: the first group contains 8 expressions with the total number of valid answers between 31 and 40; 6 expressions with 41–50 valid answers for the second group; each of the third and fourth group consists of 13 expressions with 51–60 and 61–70 valid answers, respectively; and the fifth group contains 5 expressions with 71–80 valid answers. Finally, the sixth group contains 6 expressions with a total of 81–90 valid answers (for the best- and worst performing expressions, see discussion below).

Since the database contains emotional and conversational expressions at both basic-level and subordinate level, there might be a relationship between the number of valid answers and the expression type. The results, however, do not show such a relationship: for example, subordinate emotional expressions can be found in all of the above 6 groups; the same happens for conversational expressions. The basic-level emotional expressions show a similar total number of valid answers with “sad” having the lowest number of valid answers (63), followed by “fear” with 71 valid answers, “happy” with 73 valid answers, and “disgust” with a total of 78 valid answers.

**Figure 6:**
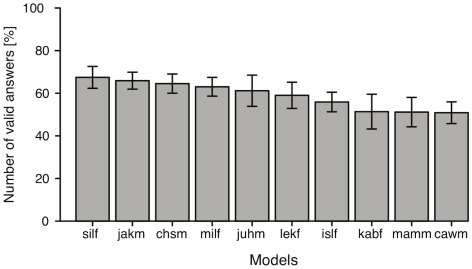
Visual condition: Mean number of valid answers per each actor. Mean number of valid answers for each of the ten models sorted in descending order. Error bars present uncorrected confidence intervals.

In order to investigate whether answer validity is similar across all ten models, we first counted the number of valid answers across all expressions for each of the ten participants and models. A repeated measures ANOVA (Greenhaus-Geisser corrected) revealed a significant effect of models on validity (*F*(9, 81) = 11.46, *p* 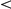.001). Hence, the number of valid answers differed significantly across the ten models. Figure 6 shows the mean number of valid answers in percent for each of the ten models. In the figure, models are sorted in descending order. Error bars represent the uncorrected confidence intervals. As can be seen in Figure 6, the largest difference between two actors is 16% (“milf” with 67% valid answers, versus “cawm” with 51% valid answers). Hence, overall, there is only a slight difference between the models concerning the number of valid answers.

**Figure 7:**
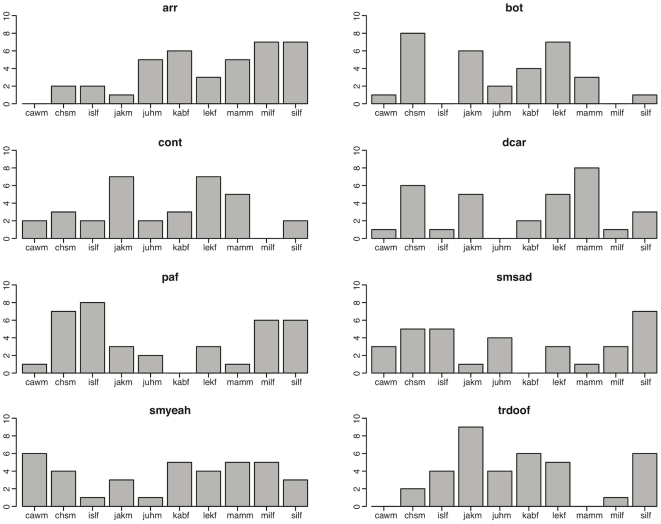
Visual condition: Frequency of valid answers for all models for worst expressions. Frequency distribution of the number of valid answers for each of the ten models for the expressions with the lowest number of valid answers for the visual condition. The abbreviations of the expressions are the following: arr = arrogant, bot = bothering, cont = contempt, dcar = don't care, paf = feeling pain, smsad = smiling nostalgic, smyeah = smiling “Yeah right!”, trdoof = doe eyed.

With respect to the number of valid answers per model for each expression, we find larger differences: Figure 7 shows the distribution of the number of valid answers per model for each of the 8 expressions for which we obtain the lowest number of valid answers. Note that for all those expressions, the overall distribution for the number of valid answer strongly differs. However, for each expression at least one model can be identified, who receives at least 6 valid answers. Thus, even for the weakest expressions there is at least one model for whom a clear correspondence between the ground-truth information - the text description - and the visually perceived expression can be found.

**Figure 8:**
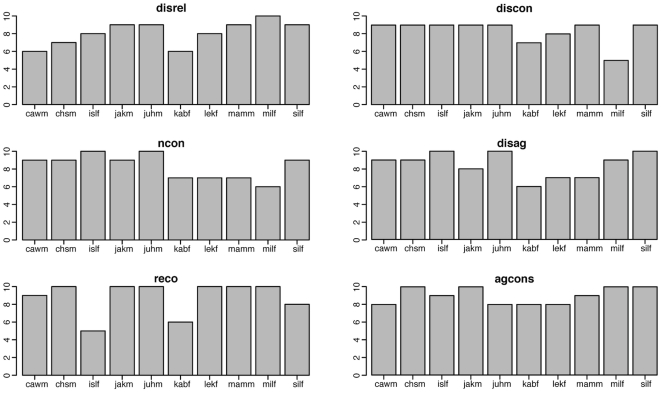
Visual condition: Frequency of valid answers for all models for best expressions. Frequency distribution of the number of valid answers for each of the ten models for the expressions with the highest number of valid answers for the visual condition. The abbreviations of the expressions are the following: disrel = reluctant disagreeing, discon = considered disagreeing, ncon = not convinced, disag = disagreeing, reco = thinking considering, agcons = considered agreeing.

The different distributions of the number of valid answers for the ten models can also be found for expressions with an overall high number of valid answers. Figure 8 shows the six expressions, for which we obtain the highest number of valid answers: “not convinced”, “thinking considering”, “agreement” and different types of “disagreement”. For example, the expression based on the text description “Someone makes a suggestion, and you hesitate.” (see the label “reco” in Figure 8), only two models have a lower number of valid answers, however, all answers for 6 models are valid. A similar pattern was found for the scenario “Someone suggests to try something. You hesitate first but then you agree.”(see the label “agcons” in Figure 8). Here, the lowest number of valid answers is found (8 out of 10) for four models. There are however, four further models for whom all answers were rated as valid.

In the context condition, we obtained ambiguous naming answers for the two scenarios (1) “Someone suggests something to you. After thinking about it you decide not to do it.” and (2) “After leaving your flat you realize you forgot to switch off the cooker.”. Interestingly, in the visual condition, we find that the corresponding expression for the first description belongs to one of the best recognized expressions. Here, out of the 100 naming answers, 81 expression labels were rated as valid. In contrast, the context condition revealed 6 out of 10 answers being valid. Here, three answers were rated as invalid by two raters and one answer only by one rater. Although four out of ten ambiguous expression names were obtained in the context condition, the visual condition points to a quite well-recognizable facial expression. A similar situation is found for the second description, for which 71 answers were rated as valid. Here, the context condition revealed 3 valid answers (for only three answers two raters agreed, the remaining were declared as invalid by only one rater). Although the visual condition showed higher naming validity for both conditions, the labels in the context condition were less ambiguous inasmuch as they included partially clear labels that have been confirmed by the visual condition.

Taking the complexity of the facial expressions in the new database into account, our analysis of the naming performance shows that the majority of expressions indeed seem to be recognizable. Subordinate and conversational expressions are distributed across levels of recognizability: some expressions can be easily recognized and while others cannot. Only the basic-level emotional expressions show similar recognizability - albeit not the best one. Moreover, for each expression we can identify at least one model for whom a high number of valid answers can be found. In general, the recognizability of expressions across models is comparable, however the distribution differs strongly between particular expressions.

*Confidence ratings:* For the confidence ratings, we obtain again a left-skewed distribution with a median of 4 and an interquartile range of 1. Participants also rated their confidence more often with the highest score (1187 cases) compared the lowest (189 cases). This overall frequency pattern was also visible for the individual confidence ratings of participants. Thus, all ten participants felt rather confident in their naming answers.

In order to analyze the distribution of confidence ratings for each of the 51 expression we calculated the frequency of confidence ratings over all participants and models separately. As with the overall data, the confidence distributions for each expressions were left-skewed as well, hence, for all expressions high confidence ratings are obtained. We found no expression for which participants felt very insecure in naming. Moreover, there is no particular model for whom low confidence ratings were obtained.

**Figure 9:**
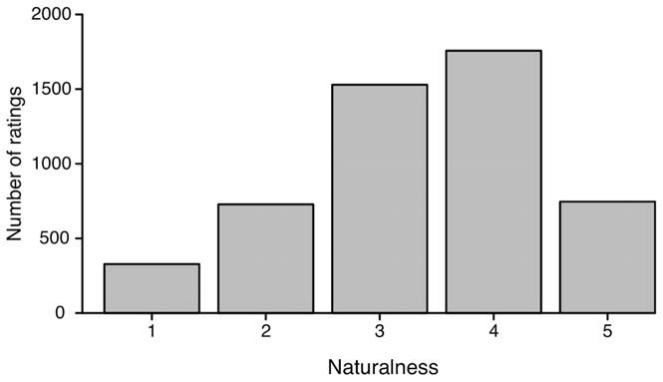
Visual condition: Frequency of naturalness scores for each expression stimulus. Frequency distribution of participants' naturalness ratings pooled over participants and expressions for the visual condition. Naturalness score 1 means “extremely posed facial expression” whereas 5 means “natural expression as it would occur during a conversation”.

*Naturalness:* In order to analyze the naturalness ratings for the facial expression “performances”, we calculated the frequency distribution for each of the 510 expression stimuli for the five rating scores across all 51 expression types, 10 models and 10 participants. The left-skewed distribution in Figure 9 shows that overall participants rated the 510 expression videos as being rather natural.

Eight participants showed overall similar distribution patterns across all expressions and models. For one participant, however, the rating of 1 was more frequently used. For the second participant, the rating scores were more equally distributed with the exception of score 1, which was comparatively less often used. Nevertheless, the vast majority of participants showed similar distribution patterns clearly biased towards the natural rating.

Investigating the naturalness ratings for each expression type by calculating the frequency of ratings across models and participants, we found that the majority of expression types again yielded left-skewed distributions (plots not shown here). However, naturalness ratings for 13 expression types were more centrally distributed. These expression types included expressions such as “aha, lightbulb moment”, “arrogant”, “fear”, “achievement”, “compassion” or different types of “thinking/remembering”. No expression type, however, had a right-skewed distribution indicating a potentially unnatural or posed expression.

In order to measure whether there is a statistical difference of naturalness ratings depending on validity type, we performed a Welch two-sample t-test. There was a significant effect for naturalness ratings with valid answers receiving higher naturalness scores than invalid answers (*t*(4307.91) = 5.82, *p* 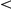.001). A post hoc power analysis, however, again revealed only a small effect size (*d* = .17).

**Figure 10:**
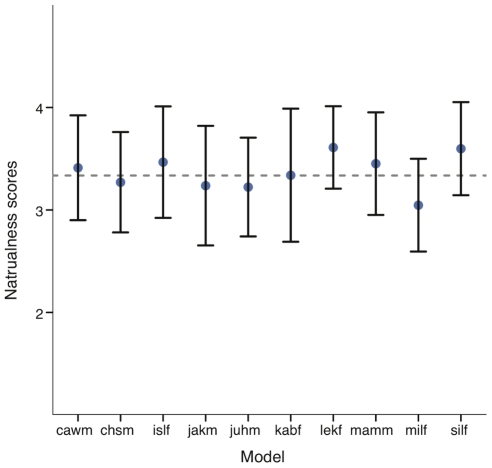
Visual condition: Average naturalness scores for each model and their corresponding confidence intervals. Mean naturalness scores for each model and their corresponding confidence intervals for the visual condition. Grey horizontal line indicates the mean naturalness ratings over all models.

While recording the database, models were not instructed on how to produce each expression allowing us to obtain rather individual expressions. Hence, there might be the possibility that some models produced the expressions in a more natural way compared to other models. We therefore calculated the mean naturalness ratings for each model and their corresponding confidence intervals (see Figure 10). If the confidence intervals of any one model would not include the grand total mean, we would find a significant difference for that model. As can be seen in Figure 10, although there was considerable variation in how models produced the expressions, the naturalness of those expressions was equally high overall.

Overall, our analysis clearly shows that expressions were rated as being “natural” by the vast majority of participants. Moreover, we were not able to find any type of expression or model that was perceived as unnatural.

#### Brief comparison between conditions

Finally, we would like to conduct a brief comparison between the first condition (based on verbal processing of context information similar to the everyday situations used during recording the new database) and the second condition (using the video sequences) based on the number of invalid answers. In the first condition, 81% of the 550 answers were rated as valid. In contrast, 60% of the 5100 answers in the second condition were rated as valid.

**Figure 11:**
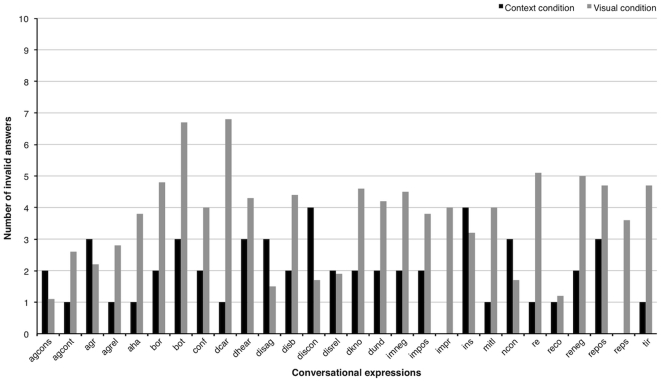
Results of the naming task for the conversational expressions in the two conditions. Plot presenting the mean amount of invalid answers for both conditions for each of the conversational facial expression. The abbreviations of the expressions are the following: agcons = considered agreeing, agcont = agree and continue, agr = agree, agrel = reluctant agreeing, aha = lightbulb moment, bor = bored, bot = bothering, conf = confused, dcar = don't care, dhear = don't hear, disag = disagreeing, dis = disbelieve, discon = considered disagreeing, disrel = reluctant disagreeing, dkno = don't know, dund = don't understand, imneg = imagine negative, impos = imagine positive, impr = impressed, ins = insecure, mitl = compassion, ncon = not convinced, re = thinking/remembering, reco = thinking considering, reneg = thinking negative, repos = thinking positive, reps = problem solving, tir = tired.

Figure 11 compares the mean number of invalid answers for conversational expressions for both conditions. Relaxing the conservative estimates of invalid answers and allowing a tolerance of 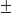
2 invalid answers, of the 28 conversational expressions, 13 expressions show no difference in the number of invalid answers. One facial expression has a lower number of invalid answers in the visual than in the context condition (“considered disagree”). In contrast, the remaining facial expressions show a considerably higher number of invalid answers compared to the descriptions in the first condition. These expressions are for example “aha, lightbulb moment”, “don't care”, “don't know”, “thinking, problem solving”.

## Discussion

One major goal of context condition was to investigate whether the context scenarios elicited clear expressions. The validation of the 55 descriptions revealed that for 50 descriptions, 7 or more naming answers were valid using a conservative validation criterion. Moreover, participants felt confident in their naming answers. Hence, we can confirm that the written descriptions can, indeed, be connected to well-defined facial expression concepts. Second, this study investigated free-naming performance for visually presented dynamic facial expressions. Our validation experiment does confirm that the vast majority of the visual presented facial expressions were recognizable. In addition, the expressions, although exhibiting a large degree of individual variation, were overall rated as being very natural.

As an alternative to a free description task, we could have chosen a forced choice method in which participants would be required to select an option from a list of expressions (or the extension to a non-forced-choice method including a “none of the above” as introduced in [95]). This method would of course enable a much cleaner analysis than a free naming task. Apart from the problem of how to deal with the larger number of categories (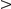
50) in such a task, an additional problem is that we are still lacking a proper facial expression “vocabulary” in order to uniquely label the expressions. With the data of the free-naming experiment presented here, we have determined such a vocabulary that might be used in future experiments - while at the same time yielding a qualitative validation measure.

Having access to the free-naming answers of the context-condition, we labeled the ground-truth information - that is the descriptions of the scenarios - according to the most frequently obtained expression name, or we tried to find generic terms that best summarize the obtained naming answers. We, however, do not claim that these are the most accurate labels, but we are confident that they do give us a good impression of the meaning of the recorded facial expressions - this is especially important for the conversational expressions, for which we have very little data so far.

Having access to a large amount of conversational expressions, it might be possible to extend existing descriptive systems of physical motion information of emotional expressions to include conversational ones as well. For such an extension, it is important to take into account the variation in performance, and whether this variation influences the recognizability [12] - our database together with the experimental data from the two validation conditions represents a first, important step into this direction.

Moreover, our aim was to create a database that contains spontaneous expressions as these expressions are most often obtained in everyday life. For recording natural and spontaneous expressions, only naive models were included in the recordings, and a “method-acting” protocol was used. In contrast to some databases based on the facial action coding system (FACS) by [96] that focus on display of action units, our models were not instructed on how to produce each expression, instead focusing on their natural performance. However, since participants were in a laboratory environment and as they also might have had to make a more conscious effort to produce the facial expressions, the degree of naturalness might have been affected. In order to capture potential differences in the perceived naturalness, the visual condition also included a rating task. It should be noted, however, that it was not possible to anchor the scale for this rating task, that is, we did not provide examples of highly unnatural or fully natural expressions, as this manipulation was not part of the database. We did make sure that participants understood about the scale ranging from posed or faked expressions on the one side to fully natural expressions (as they would happen during a conversation) on the other side. Although the resulting naturalness scale might still be highly individual, we found that the majority of participants rated the expressions as similarly natural.

Examining the naming performance and thus indirectly measuring the recognizability in the visual condition, we investigated the relationship between basic emotional and conversational expressions on the number of valid answers. Some conversational expressions yielded a higher number of valid answers. Thus, although emotional expressions are in general well recognized there seem to be further expressions with equal or even better recognizability. This is in line the theoretical work by Ekman inasmuch as the authors proposed that at least some conversational signals are easier to perform and refer to less complex subjects [12]. Comparing the naming performance of both conditions, interestingly, we find better performance for the context condition (81% versus 60% valid answers for the visual condition). Part of the reason might lie in the experimental setting: in the context condition, everyday situations were shown as text, whereas in the visual condition, 10 models illustrated each expression. These individual performances will add a certain amount of variance: some expressions might be more clearly interpretable visually than others, or, some models might be good in illustrating particular expressions whereas other models are not. Especially the latter point emphasizes the difference between eliciting an “expectation” of a clear expression (which was tested in the context condition) and the “ability” to execute it (which was tested in the visual condition). Taken together, these issues might lead one to expect a lower naming performance in the visual condition. In addition, given that the same criteria were used for determining the validity, the differences between both conditions might be due to two further reasons: missing context information in the visual condition, and different answering strategies. The context condition focuses on non-visual processing, which might have prompted a more abstract processing. However, this can also be only part of the explanation, as many expressions, indeed, show very similar results in both conditions. It might also be the case that for some conversational expression the context - such as given by the conversation, or the situation - is a crucial factor that determines recognizability; if a face appears out of nowhere, doing a facial expression, without any additional situational context (including spoken conversation, or the history just before the event), interpretation of the visual signals, might be rather difficult. Hence, on top of the individual variability, there is evidence pointing towards differences within conversational expressions with some expressions possibly begin less complex compared to emotional expressions, and some expressions depending on additional context information for better recognizability. These initial results already indicate that further research is necessary investigating the context dependency of conversational expressions.

It should also be noted that in the visual condition, only part of expressions of the entire database could be validated given that participants already performed approximately a 10 hour experiment. Since recognition accuracy for high intensity expressions is in general higher [89], we decided to first concentrate on those expressions. Further research is necessary that investigates also the low intensity expressions and extends the results to all 19 models.

### Conclusion

We created and validated a large natural database for facial expressions which allows to investigate both the emotional and the - previously little explored - conversational aspects of facial expressions. The expressions contained in the database are defined by their context taken from (conversational) situations occurring in daily life. The database consists of more than 18800 samples of video sequences from 10 female and 9 male models displaying various facial expressions recorded from one frontal and two lateral views. Each expression was recorded at two intensity levels and repeated three times in a row. We provide a detailed, consistent multi-view annotation of the database with begin-, peak-,and end-frames - information that is necessary for both perceptual and computational experiments. The database also contains auxiliary material and features, such as 3D scans and audio footage, as well as head-tracking markers worn by the models. In the following, we explore a few of the possibilities that the database and its features offer.

We envision that our database will have multiple applications in the domain of computer vision, most notably affective computing, in which the computer will automatically recognize and interpret the complex space of human facial expressions [27], [33]. The database contains 19 individuals showing 55 expressions at 2 intensities, which represents a large training and testing bed for automated recognition of complex expressions. Each expression is repeated three times by the same models, which allows for modeling of individual variability and also provides an easily accessible validation dataset for recognition purposes. In addition, the three camera views will allow computer vision researchers to test robustness of their algorithms to viewpoint changes - a factor that has rarely been taken into account in previous attempts at expression recognition. Finally, the models all wear head-bands with 6 tracking markers, which are visible in all three camera perspectives. This allows for easy, automated tracking and three-dimensional reconstruction of the rigid head motion of the models, providing access to an important signal for expression recognition. Furthermore, having access to the rigid head motion, together with fitting of a 3D scan (also contained in the database) enables easy manipulation of the video content, such as freezing of certain parts of the face, or exchanging facial parts (see for example [30], [47], [91]).

Having access to peak-frame annotations, for example, allows for investigations of possible differences in statically and dynamically displayed facial expressions. Importantly, with our database, this research can be conducted on the same set of data - note that this is not possible with the static databases due to lack of dynamic data, and also not for most dynamic databases due to lack of annotated peak frames (see Table S1). The dynamic data also allows for a detailed investigation of the temporal sensitivity of expression recognition by manipulating the speed, ordering, and number of the frames (see for example [55], [60]). The results of these studies will be important for the design of conversational agents, which can interact more naturally with humans due to production of facial expressions with natural and believable timing properties.

Moreover, as the annotations are also available for different views, view-dependent effects in encoding of facial expressions can also be examined with our database. As it has been shown by [92], face *identity* seems to be largely robust to view changes in a direct comparison task. However, relatively little is known about the effect of viewpoint on facial expression recognition, except for a short investigation by [97] and an adaption study by [98]. Investigations into this phenomenon will have an important impact for modeling of conversational avatars, for example, as modeling would need to take into account the viewpoint-dependency of facial expression processing.

The database contains recordings of the same facial expressions at two intensity levels. One of the reasons for including this factor is that it has been suggested that the general weak recognizability for spontaneous compared to posed facial expressions might be due to differences in intensity. Moreover, several studies in the different fields of expression research have examined the influence of intensity on expression recognition. For example, Sprengelmeyer and Jentzsch found a positive correlation of event-related potentials on the intensity of different expressions [99]. The study by Hess et al. revealed that the decoding accuracy of negative emotional expressions varied linearly with the physical intensity of the expressions [100]. The experimental results reported here have so far been only obtained on the high-intensity part of the database, and it will be an interesting avenue for future studies to investigate how well the results generalize also to the lower intensity stimuli. Similarly, the intensity dimension will also be interesting for testing the generalizability and robustness of computer vision algorithms.

Our database facilitates research on a large number of conversational, everyday facial expressions within a well-defined scenario context. Many databases are, for example, based on FACS, focusing on display of action units and relatively few, emotional expressions. A description of which action units make up an expression, however, is outside of FACS making it difficult to judge which elements need to go together to produce different expressions [101]. Rather than basing the expressions on a *physical* description (that is, muscle movements or action units), here we take the philosophy to base our expressions on well-defined, validated scenarios which will produce a given facial expression and which therefore constitute a *content-based* description. This content-based focus also stresses more the individual variability in producing facial expressions, as instructions and elicitation protocols are not based on constraining the movement of single muscles, but rather on emphasizing the situational context of the expression. In addition, the situational context makes it possible to distinguish different sub-ordinate expressions (such as different kinds of happiness) - something which is difficult to do using action unit descriptions. This makes our database an ideal resource in the context of affective computing, for which content-based databases containing more than just the emotional expressions are needed. In addition, the focus on conversational content in our database makes it highly suitable for investigations in the context of studies on human-to-human, and human-to-computer communication.

In summary, the MPI facial expression database provides a large test-bed for many different research fields facilitating research on a large number of conversational facial expressions.

### Obtaining the database

The new MPI database for emotional and conversational facial expressions is freely available for scientific purposes by contacting the corresponding author. An online version for accessing the database is planned.

Users will have access to each of the 55 facial expressions that were recorded by the 19 models. Three repetitions of each expression are available. We will provide the user with our pre-screening information upon request. Moreover, all expressions are available in high and low intensity as well as from three different camera perspectives. The recordings are based on single image frames by default. Users will either have access to all (uncompressed) frames, or they can also use our multi-view annotation of the frames. The expressions used in the validation experiment are also available in avi-format. In addition, all audio-recordings as well as the 3D face scans are available as wav-files and obj-files, respectively. Upon request, we will also provide additional, detailed information about the validated facial expressions as presented in the validation experiment in this paper.

## Supporting Information

Table S1 **Review of existing facial expression databases that are often used in social psycholgy.** This table lists a large variety of existing databases of facial expressions without claiming this list to be exhaustive. Note that most databases included only emotional expressions that are often based on prototypical occurrence. Moreover, there are only few databases available that video captured the expressions. For further reading about databases that concentrate on face recognition, see [102], as well as http://www.face-rec.org/databases/and http://web.mit.edu/emeyers/www/face_databases.html. For review on databases used for computational research on emotion see [44].
(PDF)

Table S2 **Summary of all facial expressions and their particular background description.** This table illustrates all recorded facial expressions and their particular everyday descriptions that can be found in the new facial expression database. The labels of the expressions as to best summarize the given free-naming answers of the validation experiment. We, however, do not claim that these are the most accurate labels, but they do give us a good impression of both the categories and the semantics of the recorded facial expressions. Note, that all facial expressions are recorded in low and high intensities and from three different camera perspectives.
(PDF)
