# Supplementary material for: The MPI Facial Expression Database — A Validated Database of Emotional and Conversational Facial Expressions
Source: PLoS One. 2012 Mar 15;7(3):e32321. doi: 10.1371/journal.pone.0032321 (PMC3305299; doi:10.1371/journal.pone.0032321)
Supplement: Table S1 — Review of existing facial expression databases that are often used in social psycholgy. This table lists a large variety of existing databases of facial expressions without claiming this list to be exhaustive. Note that most databases included only emotional expressions that are often based on prototypical occurrence. Moreover, there are only few databases available that video captured the expressions. For further reading about databases that concentrate on face recognition, see [102], as well as http://www.face-rec.org/databases/and http://web.mit.edu/emeyers/www/face_databases.html. For review on databases used for computational research on emotion see [44]. (PDF) [file pone.0032321.s001.pdf]

Table S1: Review of existing facial expression databases that are often used in social psycholgy.

Author	Face database name	Expressions[1]	Format	Short summary
[1]	GEMEP Corpus	admiration, amusement, anger, tenderness, disgust, despair, pride, shame, anxiety (worry), interest, irritation, joy (elation), contempt, panic fear, pleasure (sensual), relief, surprise, sadness	audio and video recordings	This database contains more than 7000 clips of the six basic emotions as well as subtle emotions. For the recordings 10 professional actors (5 female) were coached by a professional director. The actors received a list of the emotions together with short definitions and brief scenarios. The recordings are available in different intensity levels and part of the database has been validated.
[2]	Mind Reading: the interactive guide to emotions	expressions groups :afraid, angry, bored, bothered, disbelieving, disgust, excited, fond, happy, hurt, interested, kind, liked, romantic, sad, sneaky, sorry, sure, surprised, thinking, touched, unfriendly, unsure, wanting	Videos	The database contains over 400 videos of facial expressions that are summarized in 24 groups. Each group consists of different subordinate expressions. Each expression is displayed by 6 models ranging in age.
[3]	RU-FACS Spontaneous Expression Database	spontaneous facial actions	Videos	100 participants were asked for recording the database. Therefore, a false option paradigm was used which is though to elicit spontaneous facial expressions. Here, participants fill out a questionnaire regarding their opinions about particular social or political issues. Participants are then asked about their answer by an interviewer. Either participants are asked to tell the truth or to fool the interviewer. Moreover, participants were financially rewarded. The expressions were video captured by four synchronized cameras and clips of 33 participants have been FACS coded (onset, apex, and offset of the face action).
[4]	DaFEx	happiness, surprise, fear, sadness, anger and disgust	Videos	Comprises 1008 short videos of expressions produced by 8 Italian professional actors. Each expression was recorded in three intensities (low, medium, and high) and in two different conditions: (1) Utterance condition in which actors spoke additional sentences and (2) Non-Utterance condition. Here, actors were additionally given scenarios according to the expressions to be produced.
[5]	Montreal Set of facial displays of emotion (MSFDE)	happiness, sadness, anger, fear, disgust, and embarrassment	Images	The expressions are taken from 12 participants (European, Asian and African). Each expression was created using a direct facial action task and all expressions were FACS coded. Moreover, the expressions have been morphed into 5 different levels of intensity.
[6]	The Yale Face Database	happiness, sadness, sleepy, surprise, and wink	Images	It contains 165 greyscale images of 15 individuals one per different facial expression or configuration (with or without glasses, different camera perspectives).
[7]	University of Maryland Database	happiness, surprise, fear, sadness, anger and disgust	Videos	This database contains two sets of facial expressions: (1) The laboratory set, that includes 40 participants (varied in culture, race, and appearance) displaying their own choice of expressions. Participants were allowed to move their head without going into profile view. Moreover, they were asked to avoid speech. Each video sequence contains 1-3 expressions. (2) video recordings from TV that also contained speech.
Breidt[2]	Face Video Database of the MPI	Facial action units	Videos	The database contains videos of one actor performing approximately 45 action units which were recorded from six different viewpoints simultaneously.
[8]	Dynamic 3D FACS data set (D3DFACS)	Facial action units	Videos	For this database, between 19 and 97 different action units were recorded form 10 participants. Action unit sequences contain single and combined action units. The peak of each expression has been manually coded by certified FACS experts. Moreover, a framework is proposed that allows to build dynamic 3D morphable models for the first time.
Chen[3], 2007	Taiwanese Facial Expression Database (TFEID)	anger, contempt, disgust, fear, happiness, sadness and surprise	Images	The database consists of 7200 images captured from 40 individuals. The expressions are displayed in two (high and low) intensities and two viewing angles (0° and 45°) simultaneously.
[9]	CAFE Database	anger, disgust, happy, maudlin (for sad), fear, surprise	Images	The database consists of two normalized versions (one gamma corrected and the other histogram equalized) of the faces.

[1] Neutral expression is not included.
[2] Please see http://vdb.kyb.tuebingen.mpg.de/.
[3] Please see http://bml.ym.edu.tw/ download/html/news.htm.

[10]	Belfast Naturalistic Emotional Database	Emotional states that occur in everyday interactions as well as archetypal examples of emotions	Videos	The database consists of 298 audiovisual clips from 125 speakers. Two main sources were used for the videos: television programs and studio recordings.
[11]	FACES database	happiness, surprise, fear, sadness, anger and disgust	Images	This database aims to investigate the perception of facial expressions during lifespan. Young, middle, and older age face models were photographed. To display emotional expressions, a procedure that comprises three phases was applied prior to the photo sessions: (1) emotion induction phase that aimed triggering spontaneous experience with the respective expression, (2) personal experience phase that aimed to trigger ideosyncratical expressions, and (3) controlled expression phase in which the models underly a face trainging for each expression. Models were seated in front of a neutral background and all wore a grey shirt.
[12]	POFA (or PFA)	happiness, surprise, fear, sadness, anger and disgust	Images	The database is one of the most widely used databases, especially in cross culture studies. The photographs included in the database are based on empirical studies. The database consists of 110 frontal, grey-scale photographs of different facial expression that differ in intensity or facial configuration. Facial expressions were produced by 14 models (6 males). Models were trained for different face muscle activations associated with facial expressions. They were, however, instructed not to pose a particular emotion.
[13]	CAS-PEAL Database	smile, frown, surprise, close eyes, open mouth	Images	This database contains more than 99.000 images of over 1000 individuals with varying pose, expression, accessory, and lighting. 9 cameras, equally spaced in a horizontal semicircular circle, simultaneously captured the images across the different poses.
[14]		happiness, surprise, fear, sadness, anger and disgust	Images, however, the 3D model is dynamic in the sense that any desired angle can be selected.	The database includes Philadelphia actors (70) and actresses (69) with proportional background (Caucasian, Afrikan American, and Asian Fispanic). Each emotion was expressed under three levels of intensities and under both posed (mechanical method) and evoked (re-live appropriate experience using standard protocol) conditions. The recognizeability for some expressions were then validated in a psychophysical experiment.
[15]		angry	Images	Eight professional male actors produced the expression and more than 30 pictures per minute were taken. A preselection of the images was then shown to 21 participants who were asked to rate the emotional expression.
[16]		anger, contempt, disgust, embarrassment, fear, joy, pride, sadness, surprise	Videos	This database contains 8 professional actors. Expressions were elicited by both providing the meanings of the emotional labels and a method acting protocol. Expressions were captured by video recordings and simultaneous speech-recording. In the first condition, participants expressed the emotions vocally but without any verbal content (e.g. fear consisted of whimpering). In the second condition, participants produced speech-embedded expressions. Finally, participants also produced the facial expression in isolation.
[17]	KFDB	smile, blink, anger, surprise, and happiness	Images	Database contains expression images from 1.000 Korean participants. Overall, 7 CCD cameras were placed in a semicircle around the participant. Three imaging conditions have been considered: (1) illumination - two different light colors (fluoescent and incandescent) and eight illumination directions, (2) expressions - four different emotional expressions, and (3) poses with accessories - images were taken with and without hairband and glasses. Additional, the database includes description files (age, gender, and birth place of participant) and ground truth information (validity information and location of 26 feature points that were constantly tracked).
[18]	Indian Face Database	smile, laughter, sad/disgust	Images	The database contains greyscale images from 40 participants in different view conditions (looking up/down/right/left/towards right/left/ahead) and facial expressions.
[19]	AONE		Videos	75 mainly asian adults were asked to produce spontaneous and deliberate facial expressions. The expressions were recorded from both frontal and side views resulting in a set of 2000 video sequences of facial expressions.
[20]	CMU-Pittsburgh AU-Coded Face Expression Database	23 facial displays including single action units and combination of action units (action units represent joy, surprise, sadness, disgust, disgust, anger and fear	Videos	Facial actions were recorded from 210 adults with varying ethic background. Room lightening differed for one third of the participants. A frontal and lateral cameras were used for the recordings. Afterwards, a majority of image sequences were then FACS coded.

[21]	The Extended Cohn-Kanade (CK+) database			Extension of the CMU-Pittsburg database by increasing the number of sequences and participants. Moreover, each target expression is FACS coded and the respective emotional labels have been validated. The database also includes spontaneous sequences of smiles.
[22]	International Affective Picture System (IAPS)	Pictures varying in pleasure and arousal	Pictures	700 pictures that varied in pleasure and arousal. Each picture was rated for the degree of pleasure, arousal, and dominance.
[23]	Radboud Faces Database	anger, sadness, fear, disgust, surprise, happiness and contempt	Images	Expression pictures were taken from 49 models (39 adults and 10 children). Each expression is given in three different eye directions: straight ahead and averted to the left/right. Pictures, taken against a uniform white background, were captured from five different camera angles simultaneously. Participants were trained by FACS specialists thus based on the prototypical expressions. The database has been validated including expression judgements and ratings of the attractiveness, intensity, expression's clarity, expression's genuineness and valence.
Lundqvist[4], 1998	The Karolinska Directed Emotional Faces (KDEF)	happy, angry, afraid, disgusted, sad, surprised	Images	The database contains a set of 4900 color images taken from 70 individuals. Each expression is photographed twice from 5 different angles.
[24]	The Japanese Female Facial Expression Database	happy, angry, fear, disgusted, sad, surprised	Images	Ten female Japanese participants posed three or four examples of the facial expressions resulting in a total set of 219 images. Each actor took pictures of herself while observing herself through a semi-reflective mirror. The recordings were controlled for illumination and the visibility of expressive zones of the face. In addition, the database has been rated on emotion adjectives using a five point likert scale by 92 Japanese participants.
[25]	The AR FAce Database	smile, anger, scream	Images	More than 3000 color images of more than 100 people. Images were taken from frontal view under different illumination and occlusion conditions.
[26]	Japanese and Caucasian Facial Expression of Emotion (JACFEE)	anger, contempt, disgust, fear, happiness, sadness, and surprise	Images	The database consists of 56 different people (Japanese and Caucasian faces). Facial expressions were produced by 8 different participants. All expression faces have been FACS coded.
[27]		angry, disgust, happy, fear, sad, and surprise	Images	Color images of six children and ten adults displaying the facial expressions in two view conditions under controlled lighting: (1) frontal view, and (2) 30° to the left profile view. Before recording, models trained the expressions in front of a mirror. No explicit instructions were given as to how each emotion should be expressed.
[28]	Productive Aging Laboratory (PAL) Face Database	happy, sad, angry, annoyed, grumpy or disgusted, and surprised	Images	The database contains over 1000 grey-scale images of adults in the age of 19 to 97. Facial expression pictures are captured from a subset of participants.
[29]		agreement, disagreement, happiness, sadness, confusion, thinking, clueless, surprise, and disgust	Videos	Six different individuals (two male and four female) performed nine different expressions. Expressions were recorded from six cameras simultaneously and three times in a row. For the recordings, a method acting protocol was used. Most of the participants had no acting experience.
[30]	University of Texas Video Database	happiness, sadness, fear, disgust, anger, puzzlement, laughter, surprise, boredom, and disbelief	Videos	The database contains a total of 284 participants (208 female, 76 male). For the dynamic facial expression recordings, participants saw a 10-minute video intended to elicit different emotions. After the recording, short 5-second clips with the subject displaying the facial expressions were hand selected and included in the database along with a 5-second "blank stare" clip, which contains no facial motions but possibly other movements of the head or the eyes.
[31], [32]	MMI Facial Expression Database	single and multiple facial muscle activation and emotional expressions	Pictures and videos	The recordings of the database started in 2002 and currently comprises five different parts. 20 participants displayed a total of 31 different action units as well as two or all of the affective expressions sleepy, happy, and/or bored in part 1. Participants were instructed by FACS coder on how to produce the desired face deformation under consideration of minimizing out-of-plane head motion. The video recordings were taken from a frontal and profile view. Part 2 includes posed expressions of six emotions (happy, sad, surprise, anger, fear, and disgust) that were video recorded from 28 participants. Part 3 consists of high quality still images of 5 participants displaying all 31 action units and all emotional expressions. Part 4 contains video recordings for the spontaneous emotional expressions: happiness, surprise, and disgust. The spontaneous expressions were produced by showing 16 participants different short video clips. Sound was provided over the PC and the experimentator was present while recording assuming to elicit a higher social interaction. Part 5 contains the same expressions of 9 new participants as part 4, however, the experimentator was absent and sound was given over headphones. Part 4 is fully FACS coded whereas this is only partially the case for part 5.

[4] Please see http://www.emotionlab.se/resources/kdef.

PICS[5]	Psychological Image Collection of Stirling (PICS)	smiling, surprise, disgust	Images	PICS was developed by the University of Stirling Psychology Department and contains numerous static images of different types. Among those images are expression images of 35 individuals (18 female). For the frontal view, the expressions neutral, smiling, surprise, and disgust were captured. An image of the individuals while wearing a bathing-cap was also taken in the frontal view. The 3/4 view condition includes only the facial expressions neutral and smiling.
[33]		anger and surprise as well as further isolated expressive gestures	Images and videos	Facial expressions were recored from five different viewpoints simultaneously. Each expression was produced by eight amateur actors. The recordings were controlled for external features (like hairstyle, clothes etc.). To ensure natural expressions, actors were asked to read short stories. While imagining the stories, actors spoke out load a word fitting in the emotional context (that was "What!" for the anger expression and "Wow!" for surprised expression). Head movements were minimized.
[34]	A database of dynamic and static faces expressing highly recognizable emotions (STOIC)	happiness, disgust, fear, anger, sadness, surprise, and pain	Images and videos	Initially, the expressions were recorded from 34 participants (16 female) all of having a theatrical background. Each expression was shown in three different intensities (weak, moderate, and high). Of the initial dataset, the best 1088 movies were selected by four raters. Therefore, the criterions were: minimum body and head displacement as well as apparent authenticity of the expressed emotion. For the static version, the frame that contained the peak expression of every movie was selected. The movies of the dynamic version were reduced to 15 frames.
[35]	AT-T Database for Faces (formerly ORL database)	open/closed eyes and smile/no smile	Images	The database contains a set of 10 frontal images per each of 40 participants. Participants were seated against a dark homogenous background.
[36]	Amsterdam Dynamic Facial Expression Set (ADFES)	happiness, anger, contempt, disgust, fear, sadness, surprise, pride and embarrassment	Videos	Expressions were performed by 22 Dutch participants (12 female) with different ethnicities (12 Caucasian, 9 Mediteranean, 1 Indonesian). The expressions were recorded using a video camera and the database includes direct expressions (face forward) and an active head turn expression (face away from the camera) both filmed from two angles.
[37]	CMU Posed, Illumination, and Expression (PIE) database	smile, blink and talking	Images and video only for talking	A synchronized multi-camera system (13 cameras) was used to obtain a large variety of poses (63 participants). Additionally, a flash system was used to obtain 21 illumination conditions for two background lightings. The talking variation was video taped using 3 camera perspectives. An extension of this database, the Multi-PIE ([38]), is also available which includes more participants and additional facial expressions (at least smile and disgust).
[39]	CAFE database	happiness, sadness, surprise, anger, disgust, and fear	Images	Images of 10 participants (5 female) were captured and normalized for location of the eyes and mouth. Images are FACS coded.
Tottenham[6]	NimStim Face Stimulus Set	fearful, happy, sag, angry, surprised, calm, disgust	Images	The validated database contains a total of 646 colored facial expression stimuli captured by 70 participants (female and male) with varying ethnical background. Some participants are professional actors. Face muscles were adjusted until the desired expression was achieved.
[40]	Facial Expression and Emotion Database (FG-NET)	happiness, sadness, surprise, anger, disgust, and fear	Images together with animated image sequences	Image database contains face images showing 18 different individuals performing the basic emotions. It was tried to evoke real emotions by playing video clips or still images after a short introduction phase using a dual headed workstation. Expressions were captured at 25 frames per second.
[41]	Binghamton University 3D Facial Expression Database (BU-3DFE)	happiness, sadness, surprise, anger, disgust, and fear	Images	The database includes 100 (56 female) actors with varying ethnical background. The expressions were captured by a 3D face scanner and expressions include four levels of intensity. Associated with each expression shape model is a corresponding facial texture image captured at two views (about $\pm 45^\circ$).

This table lists a large variety of existing databases of facial expressions without claiming this list to be exhaustive. Note that most databases included only emotional expressions that are often based on prototypical occurrence. Moreover, there are only few databases available that video captured the expressions. For further reading about databases that concentrate on face recognition, see [42], as well as http://www.face−rec.org/databases/ and http://web.mit.edu/emeyers/www/face_databases.html. For review on databases used for computational research on emotion see [43].

[5] Please see http://pics.psych.stir.ac.uk/.
[6] Please see http://www.macbrain.org/resources.htm.

References

1. Bnziger T, Scherer KR (2010) Introducing the geneva multimodal emotion portrayals (gemep) corpus. In: Scherer KR, Roesch E, editors, Blueprint for affective computing: A sourcebook, Oxford University Press. pp. 166-176.

2. Baron-Cohen S, Golan O, Wheelwright S, Hill J (2004) Mind reading: The interactive guide to emotion. Jessica Kingsley.

3. Bartlett MS, Littlewort GC, Frank MG, Lainscsek C, Fasel IR, et al. (2006) Automatic recognition of facial actions in spontaneous expressions. Journal of Mutlimedia : 1-14.

4. Battocchi A, Pianesi F (2004) Dafex: Un database di espressioni facciali dinamiche. In: SLI-GSCP Workshop "Comunicazione Parlata e Manifestazione delle Emozioni". SLI-GSCP Workshop "Comunicazione Parlata e Manifestazione delle Emozioni", Padova (Italy) 30 Novembre - 1 Dicembre.

5. Beaupr M, Cheung N, Hess U (2000) La reconnaissance des expressions motionnelles faciales par des dcodeurs africains, asiatiques et caucasiens. In: Annual meeting of the Socit Qubcoise pour la Recherche en Psychologie, Hull, Quebec, Canada.

6. Belhumeur PN, Hespanha JP, Kriegman DJ (1997) Eigenfaces vs. fisherfaces: Recognition using class specific linear projection. IEEE Transactions on Pattern Analysis and Machine Intelligence, Special Issue on Face Recognition 17: 711-720.

7. Black MJ, Yacoob Y (1997) Recognizing facial expressions in image sequences using local parameterized models of image motion. International Journal of Computer Vision 25: 23-48.

8. Cosker D, Krumhuber E, Hilton A (2011) A facs valid 3d dynamic action unit database with applications to 3d dynamic morphable facial modellling. In: IEEE International Conference on Computer Vision (ICCV).

9. Dailey MN, Cottrell GW, Reilly J (2001). CAlifornia Facial Expressions (CAFE). URL `http://www.cs.ucsd.edu/users/gary/CAFE/`.

10. Douglas-Cowie E, Cowie R, Schroeder M (2003) The description of naturally occurring emotional speech. In: Proceedings of 15th International Congress of Phonetic Sciences. Barcelona, pp. 2877-2880.

11. Ebner NC, Riediger M, Lindenberg U (2010) Faces - a database of facial expressions in young, middle-aged, and older women and men: Development and validation. Behavior Research Methods 42: 351-362.

12. Ekman P, Friesen W (1976) Pictures of facial affect. Consulting Psychologists Press, Palo Alto, CA.

13. Gao W, Cao B, Shan S, Zhou D, Zhang X, et al. (2004) Cas-peal large-scale chinese face database and evaluation protocols. Technical Report JDL-TR-04-FR-001, Joint Research & Development Laboratory.

14. Gur RC, Sara R, Hagendoorn M, Marom O, Hughett P, et al. (2002) A method for obtaining 3-dimensional facial expressions and its standardization for use in neurocognitive studies. Journal of Neuroscience Methods 115: 137-143.

15. Hamm AO, Vaitl D, Lang PJ (1989) Fear conditioning, meaning, and belongingness: A selective association analysis. Journal of Abnormal Psychology 98: 395-406.

16. Hawk ST, van Kleef GA, Fischer AH, van der Schalk J (2009) "word a thousand word": Absolute and relative decoding of nonlinguistic affect vocalization. Emotion 9: 293-30.

17. Hwang BW, Byun H, Roh MC, Lee SW (2003) Performance evaluation of face recognition algorithms on the asian face database, kfdb. In: Kittler J, Nixon M, editors, Audio- and Video-Based Biometric Person Authentication. Springer Berlin / Heidelberg, volume 2688, p. 1060. doi: 10.1007/3-540-44887-X.

18. Jain V, Mukherjee A (2002). The indian face database. URL `http://vis-www.cs.umass..edu/$\sim$vidit/{I}ndian{F}ace{D}atabase/`.

19. Jiang P, Ma J, Minamoto Y, Tsuchiya S, Sumitomo R, et al. (2007) Orient video database for facial expression analysis. In: Sztandera L, editor, Intelligent Systems and Control (ISC 2007). Cambridge, MA, USA: A Scientific and Technical Publishing Company (ACTA Press), pp. 221-214.

20. Kanade T, Cohn J, Tian Y (2000) Comprehensive database for facial expression analysis. In: Fourth IEEE International Conference on Automatic Face and Gesture Recognition. pp. 46-53.

21. Lucey P, Cohn JF, Kanade T, Saragih J, Ambadar U, et al. (2010) The extended cohn-kanade dataset (ck+): A complete dataset for action unit and emotion-specifiied expressions. Computer Vision and Pattern Recognition Workshop (CVPRW) .

22. Lang P, Bradley M, Cuthbert B (1997) International affective picture system (iaps): Technical manual and affective ratings. Technical report, NIMH Center for the Study of Emotion and Attention.

23. Langner O, Dotsch R, Bijlstra G, Wigboldus DHJ, Hawk ST, et al. (2010) Presentation and validation of the radboud faces database. Cognition and Emotion : 1-12.

24. Lyons MJ, Akamatsu S, Kamachi M, Gyoba J (1998) Coding facial expressions with gabor wavelets. In: Third IEEE International Conference on Automatic Face and Gesture Recognition. IEEE Computer Society, pp. 200-205.

25. Martnez AM, Benavente R (1998) The ar face database. Technical Report 24, Computer Vision Center (CVC).

26. Matsumoto D, Ekman P (1988) Japanese and Caucasian Facial Expression of Emotion (JACFEE) and Neutral Faces (JACNeuF). [Slida]. SM Francisco. CA: Intercultural and Emotion Research Laboratory, Department of Psychology, San Francisco.

27. Mazurski EJ, Bond NW (1993) A new series of slides depicting facial expressions of affect: A comparison with the pictures of facial affect series. Australian Journal of Psychology 45: 41 47.

28. Minear M, Park DC (2004) A lifespan database of adult facial stimuli. Behavior Research Methods, Instruments & Computers 36: 630-633.

29. Nusseck M, Cunningham DW, Wallraven C, Blthoff HH (2008) The contribution of different facial regions to the recognition of conversational expressions. Journal of Vision 8: 1-23.

30. OToole AJ, Harms J, Snow SL, Hurst DR, Pappas MR, et al. (2005) A video database of moving faces and people. IEEE Transactions on Pattern Analysis and Machine Intelligence 27: 812-816.

31. Pantic M, Valstar M, Rademaker R, Maat L (2005) Web-based database for facial expression analysis. In: Proc. IEEE Intl Conf. Multimedia and Expo. pp. 317-321.

32. Valstar MF, Pantic M (2010) Induced disgust, happiness and surprise: an addition to the mmi facial expression database. In: Int'l Conf. Language Resources and Evaluation, Workshop on EMOTION. pp. 65-70.

33. Pilz KS, Thornton IM, Blthoff HH (2006) A search advantage for faces learned in motion. Experimental Brain Research 171: 436-447.

34. Roy S, Roy C, Fortin I, thier Majcher C, Berlin P, et al. (2007) A dynamic facial expression database. Journal of Vision 7.

35. Samaria F, Harter A (1994) Parameterisation of a stochastic model for human face identification. Proceedings of 2nd IEEE Workshop on Applications of Computer Vision .

36. der Schalk JV, Hawk ST, Fischer A (2009) Validation of the amsterdam dynamic facial expression set (adfes). In: International Society for Research on Emotions (ISRE), Leuven, Belgium.

37. Sim T, Baker S, Bsat M (2003) The cmu pose, illumination, and expression database. IEEE Transactions on Pattern Analysis and Machine Intelligence 25: 1615 - 1618.

38. Gross R, Matthews I, Cohn J, Kanade T, Baker S (2010) Multi-pie. Image Vision Computing : 807-813.

39. Smith ML, Cottrell GW, Gosselin F, Schyns PG (2005) Transmitting and decoding facial expressions. Psychological Science 16: 184-189.

40. Wallhoff F (2006). Facial expressions and emotion database. URL `http://www.mmk.ei.tum.de/~waf/fgnet/feedtum.html`.

41. Yin L, Wei X, Sun Y, Wang J, Rosato M (2006) A 3d facial expression database for facial behavior research. In: 7th International Conference on Automatic Face and Gesture Recognition (FGR06). Washington, DC, USA: IEEE Computer Society, pp. 211 - 216. doi:10.1109/FGR.2006.6.

42. Gross R (2005) Handbook of Face Recognition, Springer-Verlag, chapter Face Databases.

43. Cowie R, Doulgas-Cowie E, Cox C (2005) Beyond emotion archetypes: Database for emotion modelling using neural networks. Neural Networks 18: 371-388.
