# Supplementary material for: The MPI Facial Expression Database — A Validated Database of Emotional and Conversational Facial Expressions
Source: PLoS One. 2012 Mar 15;7(3):e32321. doi: 10.1371/journal.pone.0032321 (PMC3305299; doi:10.1371/journal.pone.0032321)
Supplement: Table S2 — Summary of all facial expressions and their particular background description. This table illustrates all recorded facial expressions and their particular everyday descriptions that can be found in the new facial expression database. The labels of the expressions as to best summarize the given free-naming answers of the validation experiment. We, however, do not claim that these are the most accurate labels, but they do give us a good impression of both the categories and the semantics of the recorded facial expressions. Note, that all facial expressions are recorded in low and high intensities and from three different camera perspectives. (PDF) [file pone.0032321.s002.pdf]

Table S2: Summary of all facial expressions and their particular background description.

Expression	Everyday scenario	Expression type
Agree, considered	Someone suggests to try something. You hesitate first but then you agree.	subordinate conversational
Agree, continue	During a conversation you signal your partner that you have understood everything and that he can continue.	subordinate conversational
Agree, pure	You agree with someone.	conversational
Agree, reluctant	Someone suggests something. You are really not sure about it, but in the end you agree.	subordinate conversational
Aha, Lightbulb moment	Now I get it!	conversational
Anger	One of your flat mates has taken your dinner out of the fridge which you were looking forward to eat all day long.	emotional
Arrogance/superiority	Only you are the best!	subordinate emotional
Bored	Right now you feel bored.	conversational
Annoyed	You have to do tons of work which you do not want to do at all. The night before the deadline you realize that you have to work all night long.	conversational
Confused	You lose the way in a foreign city.	conversational
Contempt	You think of someone you despise.	subordinate emotional
Don't care	Someone suggests something but you are not interested in it at all.	conversational
Didn't hear	Someone talks to you but you cannot understand it because the environment is too loud.	conversational
Disagree, pure	You disagree.	conversational
Disagree, considered	Someone suggests to try something. You hesitate first and disagree finally.	subordinate conversational
Disagree, reluctant	Someone suggests to try something. You are not up for it and finally disagree.	subordinate conversational
Disbelief	Someone tells you a true story, however, you do not want to believe it.	conversational
Disgust	You find moldy food in your fridge after you come home from a journey.	emotional
Don't know	Someone asks you for the name of the Ugandan president.	conversational
Don't understand	Someone is explaining something to you, but you do not understand it.	conversational
Embarrassment	Your professor commends your work in the seminar. You feel embarrassed.	subordinate emotional
Evasive	You ate the last piece of chocolate and hide it from the others.	conversational
Fear, "Oh my God!"	After leaving your flat you realize you forgot to switch off the cooker.	emotional
Fear, terror	A monster appears suddenly.	subordinate emotional
Happy, achievement	You have reached a goal and you are happy to have accomplished it.	subordinate emotional
Happy, laughing	You are laughing about a joke.	emotional
Happy, satiated	You are lying on your couch after a delicious dinner.	subordinate emotional
Happy, Schadenfreude	Someone whom you don't like slips on a banana peel in front of you.	subordinate emotional
Imagine, negative	You imagine something unpleasant in your future.	subordinate conversational
Imagine, positive	You imagine something pleasant in your future.	subordinate conversational
Impressed	You observe someone dancing and think: "Wow, that's really good!"	subordinate emotional
Insecurity	You use an expensive device at the University and suddenly it stops working. You are not sure if this was your fault.	conversational
Compassion	Your best friend tells you that he/she has broken up with his partner.	conversational
Maybe, not convinced	Someone suggests something but you are not really convinced.	conversational
Pain, felt	While doing sports you suddenly have an accident in which you twist one ankle and graze your knee.	subordinate emotional
Pain, seen	You watch a TV transmission of your favourite sport event. Suddenly one player has a serious accident. You can see bones sticking out of the player's body.	subordinate emotional
Annoyed, rolling eyes	You explain something for the 10th time and your listener still does not get it.	conversational
Thinking, considering	Someone makes a suggestion and you hesitate.	conversational
Thinking, remember negative	You recall an awkward situation that happened to you in the past.	subordinate conversational

Thinking, remember neutral	You think about what you had for breakfast yesterday.	conversational
Thinking, remember positive	You recall a pleasant situation that happened to you in the past.	subordinate conversational
Thinking, problem solving	You think of how old you will be in 37 months.	conversational
Sad	You fail one of your most important exams.	emotional
Smiling, endearment	A little girl smiles at you.	subordinate emotional
Smiling, encouraging	You encourage somebody by saying: "Cheer up, everything will work!"	subordinate emotional
Smiling, flirting	You are flirting.	subordinate emotional
Smiling, reluctant	A friend of you wants to go out this evening. You do not want to go with him/her. She/He tells you that a couple of other friends will also be around whom you want to see again.	subordinate emotional
Smiling, sardonic	You said a sardonic joke concerning one of your friends.	subordinate emotional
Smiling, sad/nostalgia	You recall a pleasant situation that happened to you in the past and get the feeling that everything was better back then.	subordinate emotional
Smiling, pride	You achieved something which someone didn't believe possible.	subordinate emotional
Smiling, uncertain	Something happened and you are not sure if it is positive.	subordinate emotional
Winning smile	The parents of your girlfriend visit you. You open the door and welcome them.	subordinate emotional
Smiling, "Yeah right!"	Someone tells you something incredible and you think: "Yeah, as if..."	subordinate emotional
Tired	The only thing you want to do is to lie in the bed after a long working day.	conversational
doe eyed	No everyday description available for this expression	conversational

This table illustrates all recorded facial expressions and their particular everyday descriptions that can be found in the new facial expression database. The labels of the expressions were chosen such that they best summarize the given free-naming answers of the validation experiment. We, however, do not claim that these are the most accurate labels, but they do give us a good impression of both the categories and the semantics of the recorded facial expressions. Note, that all facial expressions are recorded in low and high intensities and from three different camera perspectives.
